# Deciphering the action mechanism of the sacred plant “Ashvattha” (*Ficus religiosa* L.) in the treatment of delayed-onset muscle soreness

**DOI:** 10.1016/j.jaim.2026.101389

**Published:** 2026-08-05

**Authors:** I Gusti Agung Ayu Kartika, Kevin Awidarta, Maysaa Alakbaree, Sisca Ucche, Syazwani Itri Amran, Agung Endro Nugroho

**Affiliations:** a Yoga and Health Study Program, Faculty of Brahma Widya, Universitas Hindu Negeri I Gusti Bagus Sugriwa Denpasar, Bali, 80239, Indonesia; b Department of Pharmacology and Clinical Pharmacy, Faculty of Pharmacy, Universitas Gadjah Mada, Yogyakarta, 55281, Indonesia; c College of Biomedical Informatics, University of Information Technology and Communications (UoITC), Baghdad, Iraq; d Faculty of Science, Universiti Teknologi Malaysia, Johor Bahru, 81300, Malaysia

**Keywords:** Delayed-onset muscle soreness, *Ficus religiosa*, Network pharmacology, PTGS2, Inflammation, Molecular docking

## Abstract

**Background:**

DOMS is a common post-exercise syndrome characterized by pain, stiffness, and decreased muscle function. Conventional therapies have limited efficacy and have potential side effects.

**Objective:**

This study explored the molecular mechanisms of *Ficus religiosa*'s alleviation of DOMS using network pharmacology, molecular docking, and molecular dynamics simulation analyses.

**Materials and methods:**

This study used a network pharmacology framework to identify and analyze the phytochemicals from *F. religiosa*. Databases like IMPPAT 2.0 were used to retrieve potential compounds, while SwissTargetPrediction predicted target proteins. Therapeutic targets were identified using Genecards and OMIM databases. Protein-protein interaction networks were analyzed using STRING and Cytoscape software, while pathway enrichment was conducted using Enrichr. Molecular docking was performed using AutoDockTools (v1.5.7), followed by molecular dynamics simulations using GROMACS to validate target-compound interactions.

**Results:**

Of the 140 phytochemicals, 44 met the drug-likeness and bioavailability criteria and were associated with 39 potential therapeutic targets for DOMS. KEGG enrichment analysis revealed inflammation, oxidative stress, and immune regulation as the primary mechanisms of action. Prostaglandin-endoperoxide synthase 2 (PTGS2) was identified as a critical target. Molecular docking showed that compounds such as α-eudesmol, epi-γ-eudesmol, and α-cadinol exhibited superior binding affinities for PTGS2 compared to ibuprofen. Finally, molecular dynamics simulation demonstrated that α-cadinol exhibited the most stable binding owing to its robust hydrogen bonding, whereas epi-α-cadinol displayed progressive stabilization and efficient accommodation inside the active region of the protein.

**Conclusion:**

This study suggests *that F. religiosa* could be used as a therapeutic agent for the management of DOMS. The key compounds, α-cadinol and epi-α-cadinol, show potential anti-inflammatory and analgesic effects.

## Highlights

• Mechanisms of *Ficus religiosa* against DOMS were explored using integrated in silico approaches.
• Of 140 phytochemicals identified, 45 met drug-likeness and bioavailability criteria.
• PTGS2 emerged as a key inflammatory target associated with DOMS pathology.
• α-cadinol and epi-α-cadinol showed stronger PTGS2 binding than ibuprofen.
• *F. religiosa* showed multi-target potential against DOMS, supporting traditional use.

## 1 Introduction

Delayed-onset muscle soreness (DOMS) is a common post-exercise pain that typically occurring 12-24 h post-workout (Dehnke, 2022). The level of pain you may feel depends on the training style, intensity, frequency, and body type. DOMS can even be experienced by seasoned athletes. This pain can be felt from time to time. DOMS has been reported to occur not only in skeletal muscle but also involves collagenous connective tissue, with accumulating evidence highlighting its significant role in the underlying pathophysiology [1]. Muscle pain is reported to occur in various sports [2,3].

DOMS, a mild to moderate pain lasting 48-72 h, can affect training programs, daily activities, and competition among athletes. Immediate and practical treatments are needed, including stretching, thermal treatment and massage [4,5]. DOMS treatment can be enhanced by providing a comprehensive approach utilizing herbal medicines such as curcumin, green tea, saffron, and ginseng, which are safe and cost-effective [[6], [7], [8], [9]].

Usada Taru Premana, a traditional library used for developing herbal products, has been passed down among the Balinese people for generations. The literature mentions an herbal formula containing *F. religiosa* (Ashvattha), a sacred tree in Ayurvedic medicine, and Balinese Hindu culture to address the aches associated with DOMS. The plant has various pharmacological and ethnomedicinal properties, including antioxidant, anti-inflammatory, antidiabetic, antimicrobial, neuroprotective, and wound-healing activities [10].

*Ficus religiosa*, commonly known as Asvattha, Pipal, or Bodhidruma, has held significant religious, cultural, and medicinal importance in Indian traditions since ancient times. References to Aśvattha are found in Vedic literature, including the Atharvaveda, as well as in the Ramayana, Mahabharata, Bhagavad Gita, Upanishads, Puranas, and Buddhist texts, highlighting its sacred status and enduring cultural relevance. In Ayurveda, the plant is recognized under several synonyms that reflect its spiritual significance and therapeutic value. Traditional Ayurvedic literature describes the use of *F. religiosa* in the management of a variety of ailments, including consumption, vomiting, oral ulcers, burns, and gynecological disorders [11]. Its long-standing incorporation into classical medical systems and religious traditions underscores its importance as both a medicinal and culturally revered species.

Network pharmacology depends on high-throughput omics data analysis and network database retrieval, integrating systems biology with multidimensional pharmacology. It underscores the shift from targeting a singular protein and treatment to addressing several protein targets and therapies [12]. Network pharmacology has been extensively utilized to explore several targets and elucidate unknown mechanisms of action for diverse disorders [[13], [14], [15]].

This research employed network pharmacology to examine the active compounds of *F. religiosa* and their potential therapeutic effects on DOMS. The interactions between the active drugs and protein targets were validated by molecular docking and molecular dynamics simulations. This study may act as a reference for subsequent experiments.

## 2 Methodology

### 2.1 Network pharmacology

Our network pharmacology protocol involved seven main steps: (1) identifying potential phytochemical ingredients of *F. religiosa* from literature databases and public repositories; (2) identifying probable biological targets of the phytoconstituents of *F. religiosa*; (3) identifying known targets related to DOMS; (4) identifying potential therapeutic targets of *F. religiosa* against DOMS; (5) constructing protein-protein interactions; (6) developing gene ontology and pathway analysis; and (7) constructing a compound-target-pathway network.

#### 2.1.1 Identification and screening of phytoconstituents

Phytochemical components of the plants were collected from IMPPAT 2.0 (Indian Medicinal Plants, Phytochemistry and Therapeutics 2.0 (https://cb.imsc.res.in/imppat/)**)** and other reliable literature on related herbal ingredients. SMILES chemical notation was obtained from BindingDB (https://www.bindingdb.org/rwd/bind/chemsearch/marvin/FMCT.jsp), Chemeo (https://www.chemeo.com/), and RSCB PDB (https://www.rcsb.org/chemical-sketch). We downloaded the 2D or 3D structure of selected phytochemical components in *F. religiosa* from PubChem (https://pubchem.ncbi.nlm.nih.gov/) and/or Chemspider (https://www.chemspider.com/Search.aspx). In total, 140 phytochemical components were identified.

Bioavailability and drug likeliness (DL) are critical metrics and crucial markers for screening phytoconstituents in research. SwissADME (http://www.swissadme.ch/), PubChem (https://pubchem.ncbi.nlm.nih.gov/), and Toxtree (https://apps.ideaconsult.net/data/ui/toxtree) were used to predict ADME parameters and the overall drug-likeness score [16]. The following rules were used: molecular weight ≤500, MlogP 1-3, topological polar surface area (TPSA) ≤ 100 Å^2^, hydrogen-bond acceptors low (≤10), hydrogen-bond donors low (≤5), aromatic ring count (≤2), melting point <200 °C, and skin permeability coefficient >0.5 × 10^−3^ cm/h. The requirements were selected for the topical dosage form [17,18].

A total of 44 components satisfied the criteria. The items include (2E)-nonen-1-ol; (2E,6Z)-nonadienal; (2Z)-hexenal; (3Z)-hexenol; (3Z)-hexenyl-tiglate; (E)-cinnamyl alcohol; (E)-linalool-oxide; (E)-β-ionone; 1H-benzopyran-1-one,3,4-dihydro-8-hydroxy-3-methyl; 2-(acetylamino)hexanoic acid; 2,4-decadienal(E,E); 2,5-dihydroxybenzaldehyde; 2,6-dimethoxyphenol; 2-phenylethyl-alcohol; 4-hydroxycoumarin, allyl-caproate; benzoic-acid; benzyl alcohol; corchorifatty-acid-F; coumaran; dihydroactinidiolide; ethanol, 2-phenoxy; eugenol; linalool; n-hexanol; n-nonanal; nonanoic-acid; octanoic-acid; phenol; phenylacetaldehyde; p-vinylguaiacol; (3Z)-hexenyl-benzoate; (E)-β-ocimene; 1,2-benzenedicarboxylic-acid,mono(2-ethylhexyl)ester; dendrolasine; epi-α-cadinol; epi-γ-eudesmol; limonene; α-cadinol; α-copaene-11-ol; α-eudesmol; α-terpinene; β-eudesmol; and γ-eudesmol.

#### 2.1.2 Identification of probable biological targets of the phytoconstituents of *F. religiosa*

The SMILES of 44 screened phytoconstituents were submitted to the online target fishing web-server Swiss Target Prediction. Swiss Target Prediction (http://www.swisstargetprediction.ch/) identifies targets of tiny bioactive compounds in humans and other vertebrates. The most recent version indicates that predictions were derived by identifying analogous molecules in both two and three dimensions across an extensive array of chemicals demonstrated to be experimentally active against 3068 macromolecular targets [19]. In this investigation, the targets of *Homo sapiens* were identified, and their associated gene symbols were retrieved for further application.

#### 2.1.3 Finding known targets related to DOMS

Genecard (https://www.genecards.org/), Disgenet (https://disgenet.com/), and OMIM (https://omim.org/) were used to search the therapeutic target for DOMS. In addition to DOMS, a therapeutic target of inflammation was used in this study. DOMS is reported to have a significantly close relationship with inflammatory response [20,21].

#### 2.1.4 Identification of potential therapeutic targets of *F. religiosa* against DOMS

Potential therapeutic targets are determined from the results of combining all target proteins from all databases and removing duplicate data.

#### 2.1.5 STRING protein-protein interaction

STRING is a database that compiles both established and anticipated protein-protein interactions. The recognized “potential therapeutic targets of *F. religiosa* for DOMS” were submitted to STRING (https://string-db.org/) to create the Protein-Protein Interaction network at a medium threshold (0.40) with the species specified as ‘Human.' This was used to establish intercommunicative protein networks. The PPI network was imported into Cytoscape 3.9.1 for examination of topological characteristics.

#### 2.1.6 GO and KEGG pathway enrichment analysis

We conducted Gene Ontology (GO) and KEGG enrichment analyses utilizing Enrichr (https://maayanlab.cloud/Enrichr/) to further examine the functional annotation and pathways associated with targets relevant to DOMS.

#### 2.1.7 Construction of compound-target-pathway network

Interaction data, including the *F. religiosa*-Pubchem ID (PCID), PCID-target, and target-pathways, were compiled. The network was developed via Cytoscape 3.9.1 software, based on the three interaction datasets. CytoHubba, a Cytoscape plugin for network analysis, was used to locate gene hubs.

### 2.2 Molecular docking method

The re-docking process started by downloading the target protein from the RSCB PDB database in the ∗. pdb format. The protein was then uploaded into the Chimera software so that the native ligand could be separated from the protein and both could be saved in the same format. AutoDockTools (v1.5.7) software was used to dock the native ligand with the target protein. A grid box of size 40x40x40 (grid spacing 0.375 Å) was used to match the position of the active binding site of the native ligand, and all other parameters were left alone. Good re-docking results were expected with an RMSD value of <2 Å. We began the docking procedure by searching the PubChem database for 13 compounds with potential anti-inflammatory properties to obtain SMILES. These compounds were then uploaded into the MarvinSketch software for protonation optimization (major microspecies), aiming to obtain a structure that aligns with the body's pH of 7.4. Furthermore, we performed ligand energy optimization using the conformation (conformers) feature in MarvinSketch software to determine the lowest energy ligand conformation. We then saved the ligand conformation in Tripos mol2 format (∗. mol2), uploaded into BIOVIA Discovery Studio software, separated the lowest-energy ligand conformation, and saved it in (∗. pdb) format. The target protein in ∗.pdb format was obtained from the RSCB PDB database and subsequently uploaded into Chimera software to facilitate the separation of the native ligand. The AutoDockTools (v1.5.7) software was employed to dock the test ligand with the target protein utilizing a grid box of 40x40x40 (grid spacing of 0.375 Å), informed by the outcomes of the re-docking validation. All other parameters remained the same. We then analyzed the docking results using the BIOVIA Discovery Studio software.

### 2.3 Molecular dynamics simulation

The simulations were performed for 100 ns utilizing the GROMACS 2018.8 package with the GROMOS 96 54a7 force field to evaluate the stability of the protein-ligand complexes. The simulations were conducted on a system equipped with an 8-core Intel Core i7-9700F processor and an NVIDIA GeForce RTX 2080 Ti GPU, utilizing the Ubuntu 18.04.6 LTS operating system. Protein preparation was conducted with the pdb2gmx program, whereas ligand topology files for the ligand molecules were produced using the Automated Topology Builder (ATB) and Repository (version 3.0; https://atb.uq.edu.au/). Each system was situated within a cubic enclosure measuring x = 8.976, y = 11.412, z = 8.233, with a volume of 3018.13 nm^3^. Solvation was conducted with simple point-charge (SPC) water molecules, with the addition of sodium ions for charge neutralization via the genion module. The systems were subjected to energy minimization to achieve 1000 kJ, followed by temperature equilibration at 300 K via a two-step ensemble technique (NVT and NPT) [22], each lasting 100 ps. During the NVT phase, Berendsen thermostats without pressure coupling were employed, whereas the Parrinello-Rahman method was utilized in the NPT phase to sustain a pressure of 1 bar. The long-range electrostatics were computed utilizing the Particle Mesh Ewald method. Multiple analyses, including as RMSD, RMSF, radius of gyration (Rg), and hydrogen bonding, were conducted utilizing the GROMACS software program. Trajectory data were evaluated and visualized utilizing GROMACS for computational assessment and XMGrace software for graphical representation.

## 3 Results

### 3.1 Network pharmacology

#### 3.1.1 Identification and screening of phytoconstituents

The study searched multiple databases for information on *F. religiosa*, but only found information on IMPPAT 2.0. To access more recent data, compound data from scientific articles have been added. A total of 140 phytochemical components were collected from *F. religiosa*, of which 44 met the bioavailability and drug-likeness criteria. Filtering of these parameters predicted the local skin absorption and pharmacological activity of these compounds.

#### 3.1.2 Identificaton of potential biological targets of the phytoconstituents of *F. religiosa*

Swiss Target Prediction was used to analyze 44 phytoconstituents, generating 4495 protein target predictions. After eliminating duplicates, 778 distinct targets were identified and used as protein target data for *F. religiosa*.

#### 3.1.3 Identification of known therapeutic targets of DOMS

DOMS is a distinct condition that operates on a pathological mechanism different from that of muscle soreness. From several databases searched, 26 target proteins for DOMS were found only using GeneCards®, the Human Gene Database (https://www.genecards.org/). They are Catechol-O-Methyltransferase (COMT), Myoglobin (MB), Superoxide Dismutase 1 (SOD1), Desmin (DES), BDNF Antisense RNA (BDNF-AS), Myeloperoxidase (MPO), Elastase, Neutrophil Expressed (ELANE), C–C Motif Chemokine Ligand 2 (CCL2), Catalase (CAT), Choline Kinase Alpha (CHKA), C–C Motif Chemokine Receptor 2 (CCR2), NAD(P)H Quinone Dehydrogenase 1 (NQO1), SOD2 Overlapping Transcript 1 (SOD2-OT1), Interleukin 6 Receptor (IL6R), Glucuronidase Beta (GUSB), Integrin Subunit Alpha M (ITGAM), Myosin Heavy Chain 7 (MYH7), Filamin C (FLNC), Tripartite Motif Containing 63 (TRIM63), Fc Gamma Receptor Ia (FCGR1A), Actinin Alpha 3 (ACTN3), Myotilin (MYOT), Obscurin, Cytoskeletal Calmodulin And Titin-Interacting RhoGEF (OBSCN), Troponin I1, Slow Skeletal Type (TNNI1), Myozenin 1 (MYOZ1), and IGF2 Antisense RNA (IGF2-AS). Two hundred target proteins of inflammation from OMIM (https://omim.org/) were added to enrich the data on target proteins in this study.

A total of 224 protein targets were acquired following the elimination of duplicate data. IL6R (Interleukin-6 receptor) dan FCGR1A (Fc fragment of IgG receptor Ia) were the targets mentioned in both sources. IL6 is an inflammatory cytokine that exerts several biological effects. Research has demonstrated a critical role of neuroinflammation in the development of pathological pain [23]. A protein that is crucial for the immunological response is encoded by FCGR1A. Immunological microenvironment changes in DOMS [24].

#### 3.1.4 Identification of potential therapeutic targets of *F. religiosa* against DOMS

**Fig. 1:**
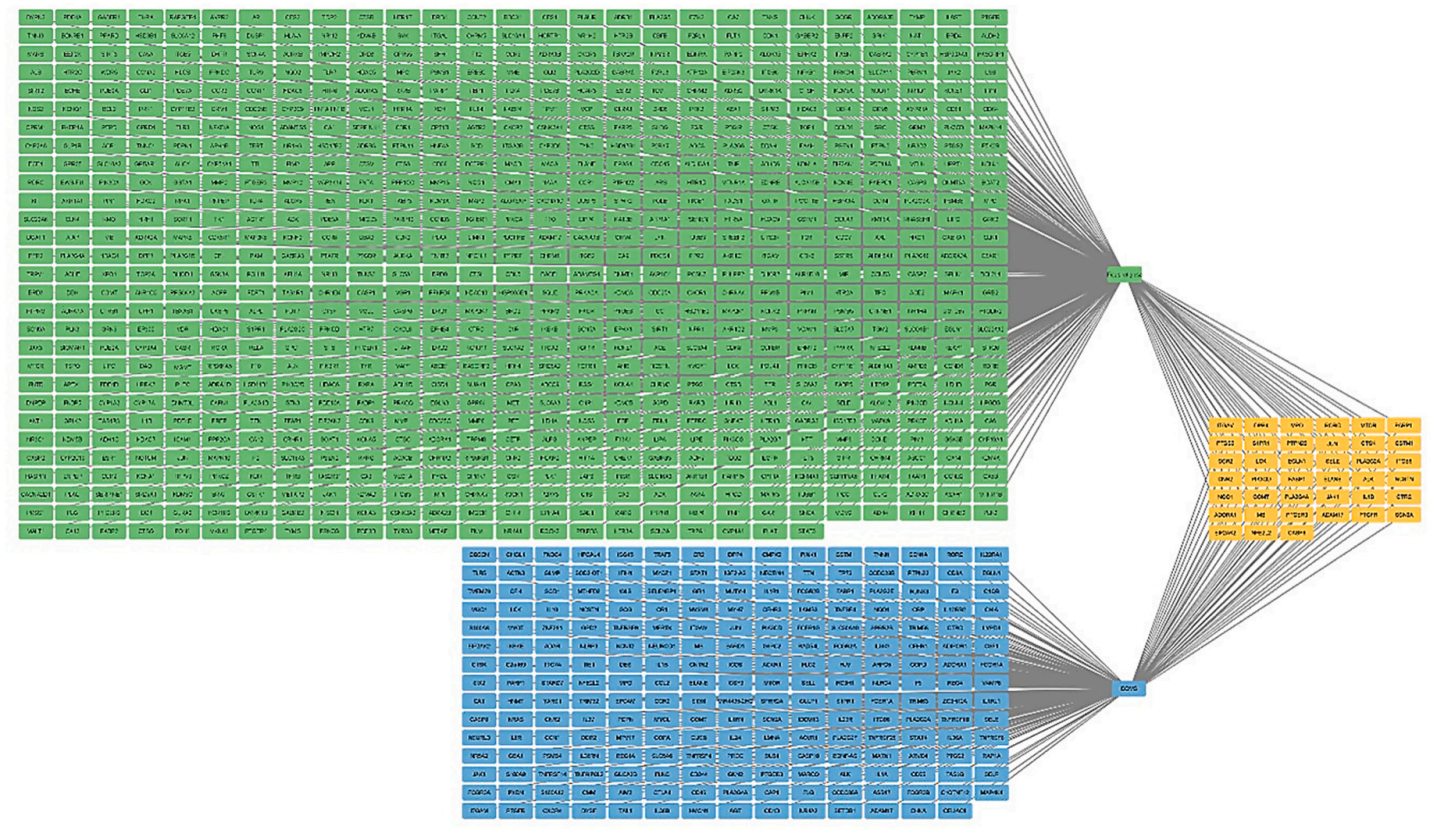
Venn diagram for DOMS therapeutic targets.

The intersection of target proteins from DOMS and *F. religiosa* is identified as the “potential therapeutic targets of *F. religiosa* against DOMS” and will be subjected to additional analysis. Thirty-nine targets were identified, including PTGER3, EIF2AK2, CNR2, PTPN22, GSTM1, ELANE, CTRC, FABP1, MB, NQO1, DPP4, PLA2G2A, S1PR1, ALK, ADORA1, CASP8, PTGFR, SELE, NFE2L2, MTOR, LCK, CCR2, PLA2G4A, ITGB6, MPO, JAK1, ADAM17, ITGAV, EGLN1, RORC, PTGS2, IL1B, NCSTN, CTSK, PIK3CD, JUN, SCN9A, COMT, and PARP1 (Fig. 1). The detailed data underlying Fig. 1 are provided in Supplementary Table 1 for clarity.

#### 3.1.5 STRING protein-protein interaction

**Fig. 2:**
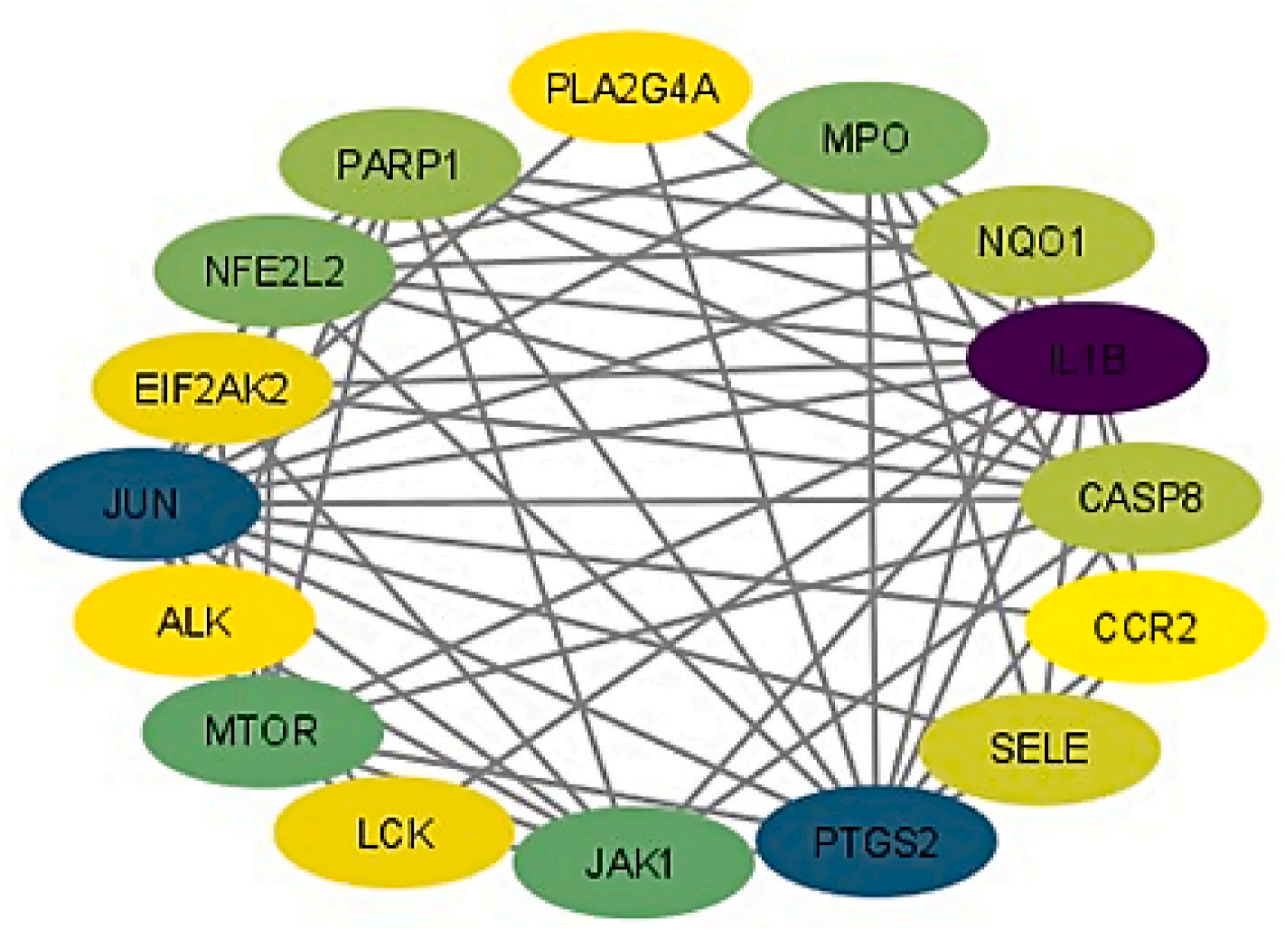
STRING protein-protein interaction network. The alteration of the node color from yellow to purple signifies an increase in the degree value. (For interpretation of the references to color in this figure legend, the reader is referred to the Web version of this article.)

The PPI network (Fig. 2), comprising 34 nodes and 102 edges, illustrated the proteins and their projected functional connections. The top 16 possible therapeutic targets with a degree of at least 5 were chosen for further investigation based on network analysis (Table 1).

**Table 1:** STRING PPI Network analysis report.

No	Gene	Protein Name	Degree	Stress	Betweenness Centrality	Closeness Centrality
1	IL1B	Interleukin-1, beta	23	856	0.38	0.73
2	PTGS2	Prostaglandin-endoperoxide synthase 2 (prostaglandin G/H synthase and cyclooxygenase)	17	548	0.21	0.65
3	JUN	Jun protooncogene, AP-1 transcription factor subunit	17	500	0.16	0.65
4	MTOR	Mechanistic target of rapamycin	11	182	0.04	0.55
5	JAK1	Janus kinase 1 (a protein-tyrosine kinase)	11	178	0.04	0.54
6	NFE2L2	Nuclear factor, erythroid-derived 2-like 2	10	140	0.03	0.56
7	MPO	Myeloperoxidase	10	156	0.05	0.55
8	PARP1	Poly(ADP-ribose) polymerase 1	9	88	0.02	0.55
9	CASP8	Caspase 8, apoptosis-related cysteine protease	8	12	0.00	0.52
10	NQO1	NAD(P)H Quinone Dehydrogenase 1	8	354	0.12	0.54
11	SELE	Selectin E (endothelial leukocyte adhesion molecule-1)	7	100	0.03	0.52
12	EIF2AK2	Eurkaryotic translation initiation factor 2-alpha kinase 2	6	4	405844155844.16	0.49
13	CCR2	C–C Motif Chemokine Receptor 2	6	18	0.00	0.5
14	LCK	Lymphocyte-specific protein tyrosine kinase	6	66	0.01	0.49
15	ALK	Anaplastic lymphoma kinase (Ki-1)	5	30	0.00	0.43
16	PLA2G4A	Phospholipase A2, group IVA, cytosolic	5	18	0.00	0.49

The PPI network was analyzed using four centrality indices: degree, betweenness, proximity, and stress. These indices are crucial in graph theory for subnetwork analysis and identifying key proteins linked to a disease. The degree (k) measures the interaction between proteins, stress centrality (SC) quantifies the number of shortest routes between points, betweenness centrality (BC) measures the node's role as a conduit, and closeness centrality (CC) measures the mean length of the shortest path to reach all proteins. A higher CC value indicated a more central protein. Proteins with the highest degree were used for further analysis to prevent spurious nodes or interactions [25].

#### 3.1.6 GO and KEGG pathway enrichment analysis

The research discovered 20 enriched KEGG pathways with a p-value cutoff of under 0.01 (Table 2). A overview of the KEGG 2021 human pathways was produced utilizing SRPlot (https://www.bioinformatics.com.cn/en). The Gene Ontology functional enrichment analysis was performed via Enrichr, resulting in the generation of a bubble chart depicting the enriched pathways of the genes of interest. The bubble chart illustrates the KEGG pathway name and rich factor (the ratio of recognized input genes to the total number of genes annotated in that pathway) on the X-axis. The size and color of the bubble represent the number of input genes annotated in the pathway and the negative logarithm base 10 of the P-value, respectively. The data are included in the Supplemental Files.

**Table 2:** KEGG 2021 human score.

No.	Pathway Name	Genes of Interest Involved	p-value
1	Necroptosis	CASP8, PARP1, IL1B, EIF2AK2, PLA2G4A, JAK1	0.0000000017
2	Pathways in cancer	CASP8, PTGS2, MTOR, JAK1, NFE2L2	0.0000000025
3	Kaposi sarcoma-associated herpesvirus infection	JUN, CASP8, EIF2AK2, PTGS2, MTOR, JAK1	0.0000000055
4	PD-L1 expression and PD-1 checkpoint pathway in cancer	ALK, JUN, LCK, MTOR, JAK1	0.0000000065
5	Th17 cell differentiation	JUN, LCK, IL1B, MTOR, JAK1	0.0000000166
6	TNF signaling pathway	JUN, CASP8, IL1B, PTGS2, SELE	0.0000000209
7	Fluid shear stress and atherosclerosis	NQO1, JUN, IL1B, SELE, NFE2L2	0.0000000620
8	Measles	JUN, CASP8, IL1B, EIF2AK2, JAK1	0.0000000620
9	Leishmaniasis	JUN, IL1B, PTGS2, JAK1	0.0000003568
10	Lipid and atherosclerosis	JUN, CASP8, IL1B, SELE, NFE2L2	0.0000005435
11	Human cytomegalovirus infection	CASP8, IL1B, PTGS2, MTOR, JAK1	0.0000006805
12	IL-17 signaling pathway	JUN, CASP8, IL1B, PTGS2	0.0000007974
13	C-type lectin receptor signaling pathway	JUN, CASP8, IL1B, PTGS2	0.0000011970
14	NF-kappa B signaling pathway	PARP1, LCK, IL1B, PTGS2	0.0000011970
15	Osteoclast differentiation	JUN, LCK, IL1B, JAK1	0.0000026600
16	Human papillomavirus infection	CASP8, EIF2AK2, PTGS2, MTOR, JAK1	0.0000045290
17	Alzheimer disease	CASP8, IL1B, EIF2AK2, PTGS2, MTOR	0.0000076870
18	Influenza A	CASP8, IL1B, EIF2AK2, JAK1	0.0000085680
19	NOD-like receptor signaling pathway	JUN, CASP8, IL1B, JAK1	0.0000108500
20	Epstein-Barr virus infection	JUN, CASP8, EIF2AK2, JAK1	0.0000167100

The objective of functional enrichment analysis is to identify specific biological annotations associated with a gene set, where a higher GO level signifies more precise functional annotations [26]. The Kyoto Encyclopedia of Genes and Genomes (KEGG) provides a repository for the systematic examination of gene activity via gene and molecular networks [27]. Analysis of protein targets indicates that *F. religiosa* compounds, via their binding to target proteins, can influence various biological pathways, particularly the NF-kappa B and TNF signaling pathways associated with the inflammatory process [28,29]. The results of the enriched biological processes for potential target genes indicate that the target of the *F. religiosa* compound is associated with the regulation of the defense response. DOMS is characterized by intense discomfort, commonly linked to acute inflammation. This defensive response encompasses blood vessels, immune cells, and chemical mediators, which are believed to be the principal catalysts of cellular damage and facilitate tissue repair [30].

#### 3.1.7 Construction of compound-target-pathway network

**Fig. 3:**
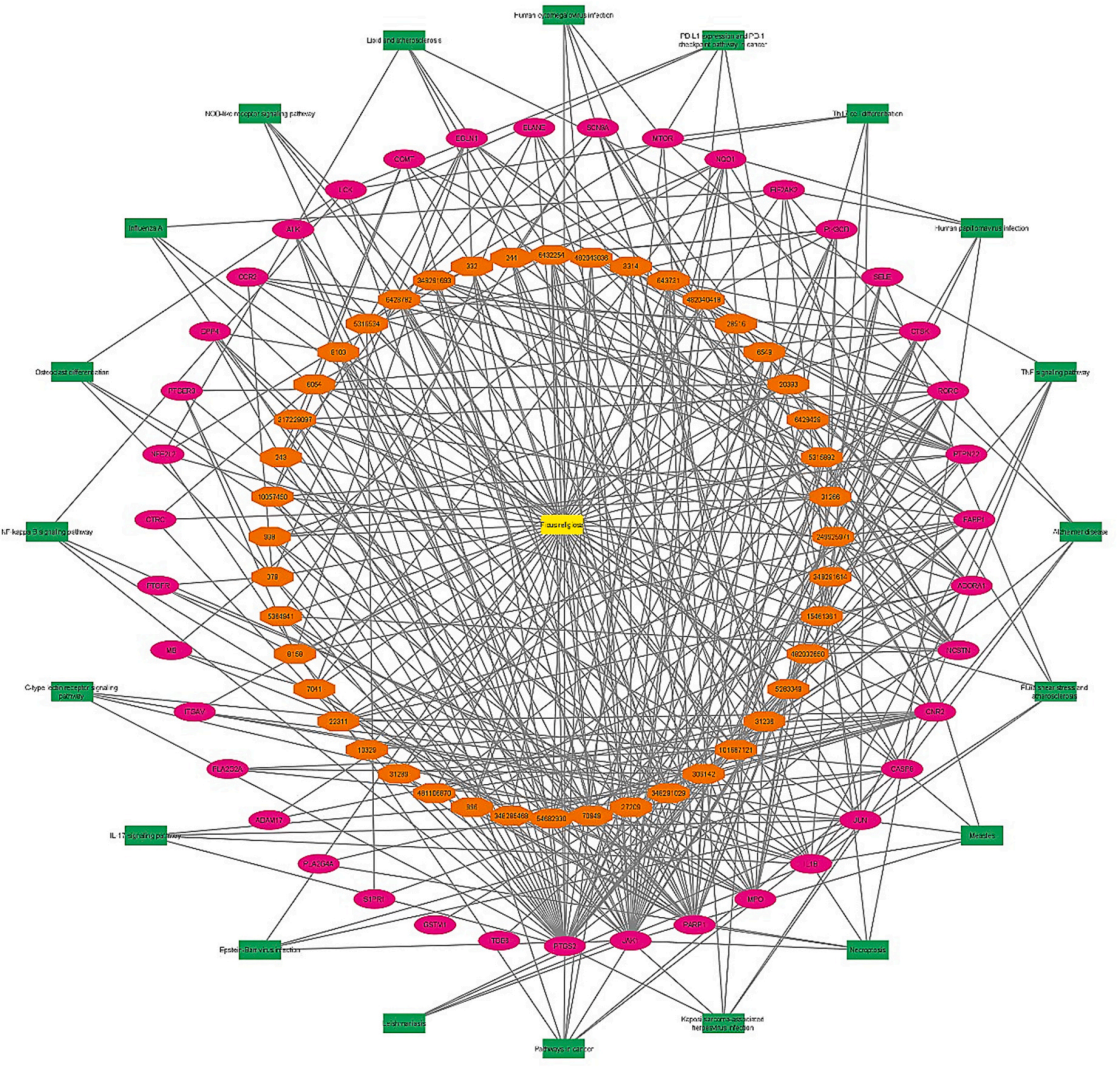
The network architecture was depicted utilizing Cytoscape 3.9.1 software to illustrate the relationships among phytochemical substances, common targets, DOMS-related targets, and pathways. The nodes signify chemicals, targets, and pathways, while the edges denote interactions.

The network comprises a chemical, a target, and a pathway, as seen in Fig. 3. The nodes delineate the phytoconstituents, prevalent targets, and pathways. Network topological metrics were evaluated, and 10 principal hub targets (Table 3) were identified for further evaluation. Supplementary Tables 2, 3, and 4 provide the detailed input data for Fig. 3.

**Table 3:** Analysis report of the Compound-Target-Pathway Network displaying the top 10 hub protein targets (Degree ≥Average value).

No	Gene Symbol	Gene Name	Degree	Betwenness	Closeness
1	PTGS2	Prostaglandin-endoperoxide synthase 2 (prostaglandin G/H synthase and cyclooxygenase)	37.0	2043.75	64.75
2	JAK1	Janus kinase 1 (a protein-tyrosine kinase)	33.0	1672.35	60.58
3	PARP1	Poly(ADP-ribose) polymerase 1	26.0	735.00	56.67
4	MPO	Myeloperoxidase	16.0	2218.51	49.25
5	IL1B	Interleukin-1, beta	16.0	310.83	46.50
6	CASP8	Caspase 8, apoptosis-related cysteine protease	15.0	274.46	45.58
7	JUN	Jun protooncogene, AP-1 transcription factor subunit	15.0	200.77	45.83
8	CNR2	Cannabinoid receptor 2	14.0	77.35	46.92
9	NCSTN	Nicastrin	13.0	262.73	44.75
10	ADORA1	Adenosine A1 receptor	13.0	105.55	48.50

Prostaglandin-endoperoxide synthase 2 (PTGS2), commonly referred to as cyclooxygenase, is the principal enzyme involved in prostaglandin biosynthesis, functioning as both a peroxidase and a dioxygenase. JAK1 is crucial for modulating the expression of genes associated with inflammation, epithelial remodeling, and the advancement of metastatic cancer. This gene is essential in the interleukin-6 (IL-6)/JAK1/STAT3 immunological and inflammatory pathways and functions as a therapeutic target for alleviating cytokine storms. PARP1 has been associated with multiple intracellular metabolic pathways and is recognized for its impact on muscular soreness [31]. This gene is crucial for the commencement of many DNA repair mechanisms. MPO and its resultant oxidants contribute to disease pathogenesis by oxidizing biomolecules, which promotes inflammation and oxidative stress. Certain researchers have discovered that MPO insufficiency or the use of MPO inhibitors may mitigate inflammation and tissue damage. IL-1β is a pro-inflammatory cytokine linked to pain, inflammation, and autoimmune diseases [32]. CASP8 governs inflammasome activation and the production and maturation of pro-inflammatory cytokines IL-1 and IL-18, therefore controlling inflammation independently of death receptor signaling [33]. JUN, a transcription factor, is phosphorylated subsequent to JNK activation. Diverse stresses, including as physical exertion and injury, can activate JNK. JNK may contribute to the molecular and cellular changes in human skeletal muscles during injury-inducing exercise [34]. The Cnr2 receptor is linked to cancer, bone metabolism, and pain perception. NCSTN encodes a type I transmembrane glycoprotein that is a crucial component of the multimeric γ-secretase complex. NCSTN is recognized for its function in facilitating cell proliferation and metastasis. Consequently, NCSTN may serve as a predictive biomarker and therapeutic target for cancer therapy [35]. The concluding entry was ADORA1. This gene is expected to enable many tasks, including G protein-coupled adenosine receptor activity, G-protein beta/gamma-subunit complex binding activity, and heterotrimeric G-protein binding activity. It functions in the modulation of sensory perception associated with pain. All ten protein targets, except NCSTN, have potential associations with DOMS therapy for both inflammation and pain management.

### 3.2 Molecular docking

Based on the primary hub target proteins identified in the STRING PPI network (Table 3) and the evaluation of the efficacy of target proteins and compounds in the inflammatory process as per existing literature, the most prominent protein target, PTGS2, was docked with its corresponding standard inhibitors, the conventional drug DOMS (Ibuprofen), and various compounds from *F. religiosa*. Table 4 displays the docking scores. The 3D docking configurations and 2D ligand–protein interaction visuals of the target, including its relevant standard, conventional medication, and phytoconstituents demonstrating optimal binding affinity, are included in the Supplemental Files.

**Table 4:** Binding affinity of FR compounds on PTGS2.

Compounds	PubChem ID	Docking score	Interacting residues
Native ligand	-	−3.79	Arg438, Glu486, Glu490, Arg185, Lys180, Leu183, Glu179, Arg184
Ibuprofen	3672	−4.11	**Leu183**, Pro442, **Arg184**, **Arg185**, **Arg438**, **Glu179**
4-Hydroxycoumarin	54682930	−3.60	-
Linalool	6549	−2.84	-
(E)-Cinnamyl alcohol	5315892	−3.20	-
(E)-Linalool-oxide	6432254	−2.98	-
(E)-β-Ionone	348291614	−3.67	-
Eugenol	3314	−3.07	-
Octanoic acid	379	−2.74	-
epi-γ-Eudesmol	6428429	−4.32	Glu179, Lys180, Leu183, Arg184, Pro442, Arg185
epi-α-Cadinol	348291693	−4.28	Arg185, Arg184, Val440, Pro442, Leu183, Glu179, Lys180
α-Cadinol	317229097	−4.29	Arg185, Arg184, Val440, Pro442, Leu183, Glu179, Lys180
α-Eudesmol	482043036	−4.38	Arg185, Arg184, Pro442, Leu183, Glu179, Lys180
γ-Eudesmol	482032650	−4.18	Arg185, Lys186, Arg184, Leu183, Glu179

The docking results showed that there are five *F. religiosa* compounds that have better binding affinity values compared to the native ligand and ibuprofen as standard drugs, such as α-eudesmol (-4.38), epi-γ-Eudesmol (-4.32), α-cadinol (-4.29), epi-α-cadinol (-4.28), and γ-eudesmol (-4.18). Compounds with stronger binding affinities are more effective in inhibiting or regulating PTGS2, potentially modulating its activity in inflammatory processes. Differences in binding affinities can be attributed to factors such as structural and physicochemical properties of the compounds and their interactions with the PTGS2 enzyme-binding site.

### 3.3 Molecular dynamics simulation analysis

**Fig. 4:**
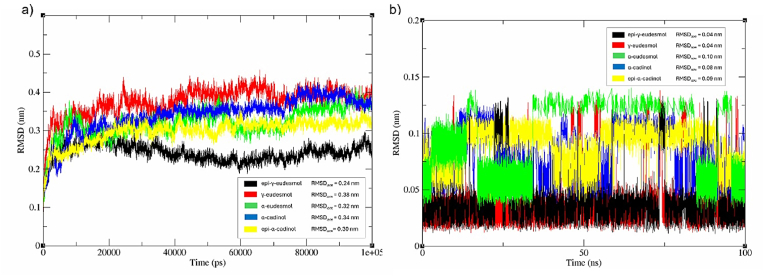
(a) Backbone RMSD of ligand-protein complexes and (b) Ligand RMSD within the protein binding site over 100 ns.

Molecular dynamic simulations were conducted on each ligand-protein complex to assess stability, conformational changes, and binding affinity differences among the five selected ligand molecules over a 100 ns simulation. Root Mean Square Deviation (RMSD) data were calculated to analyze system equilibration and stability trends. Fig. 4a illustrates that all trajectories displayed RMSD values between 0.1 nm and 0.5 nm, indicative of stable ligand-protein systems.

**Fig. 5:**
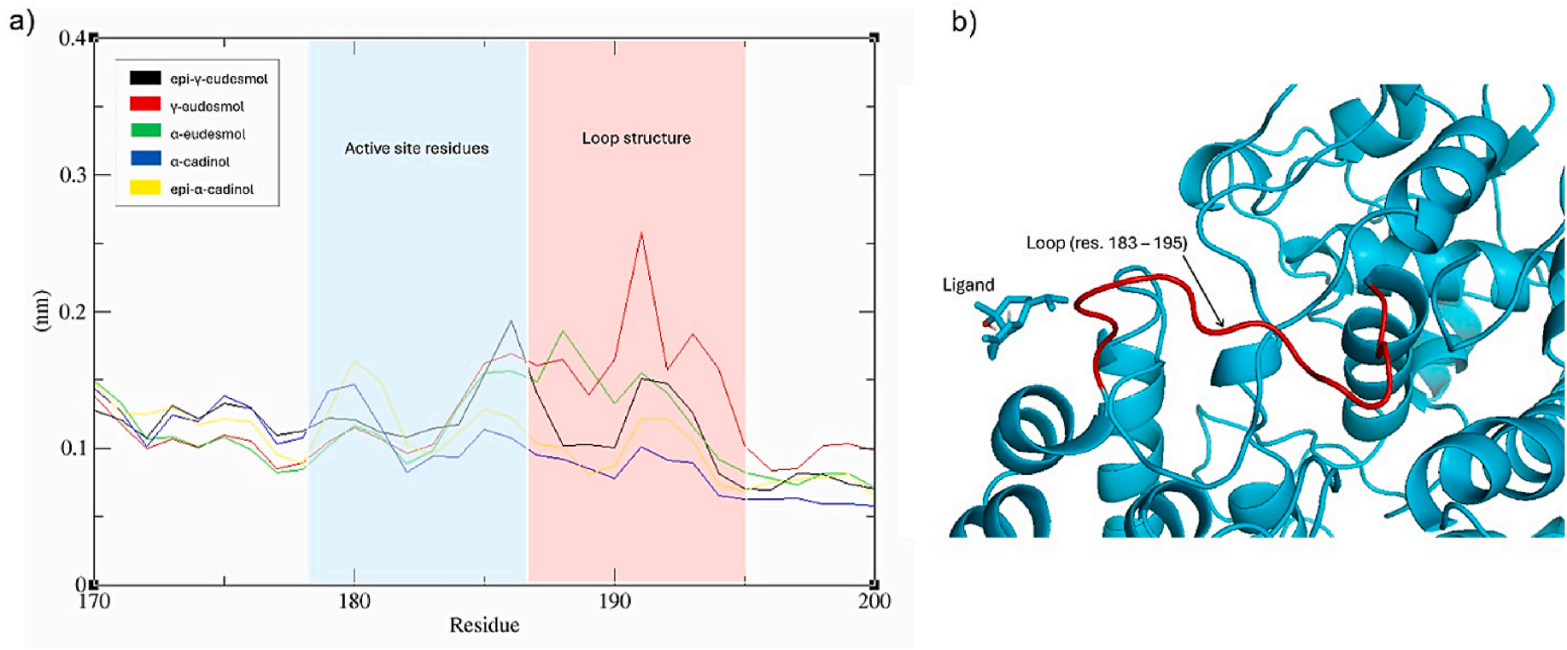
a) Root Mean Square Fluctuation (RMSF) of interacting residues within the protein active site and b) Visualization of the corresponding flexible loop region (in red) in the ligand binding site. (For interpretation of the references to color in this figure legend, the reader is referred to the Web version of this article.)

Additionally, the ligand RMSD values (Fig. 4b) were analyzed to evaluate binding stability within the active site of the protein. Most ligands stabilized within the first 20 ns, with RMSD values fluctuating between 0.02 nm and 0.15 nm, suggesting stable ligand conformations without significant deviations. Root Mean Square Fluctuation (RMSF) analysis (Fig. 5) further supported this finding, showing low fluctuations between residues 179 and 186 across all ligand-bound states. However, the higher fluctuations between residues 183 and 195 corresponded to a flexible loop region, as shown in Fig. 5b. These results suggested that the selected ligands maintained stable binding interactions with the protein.

**Fig. 6:**
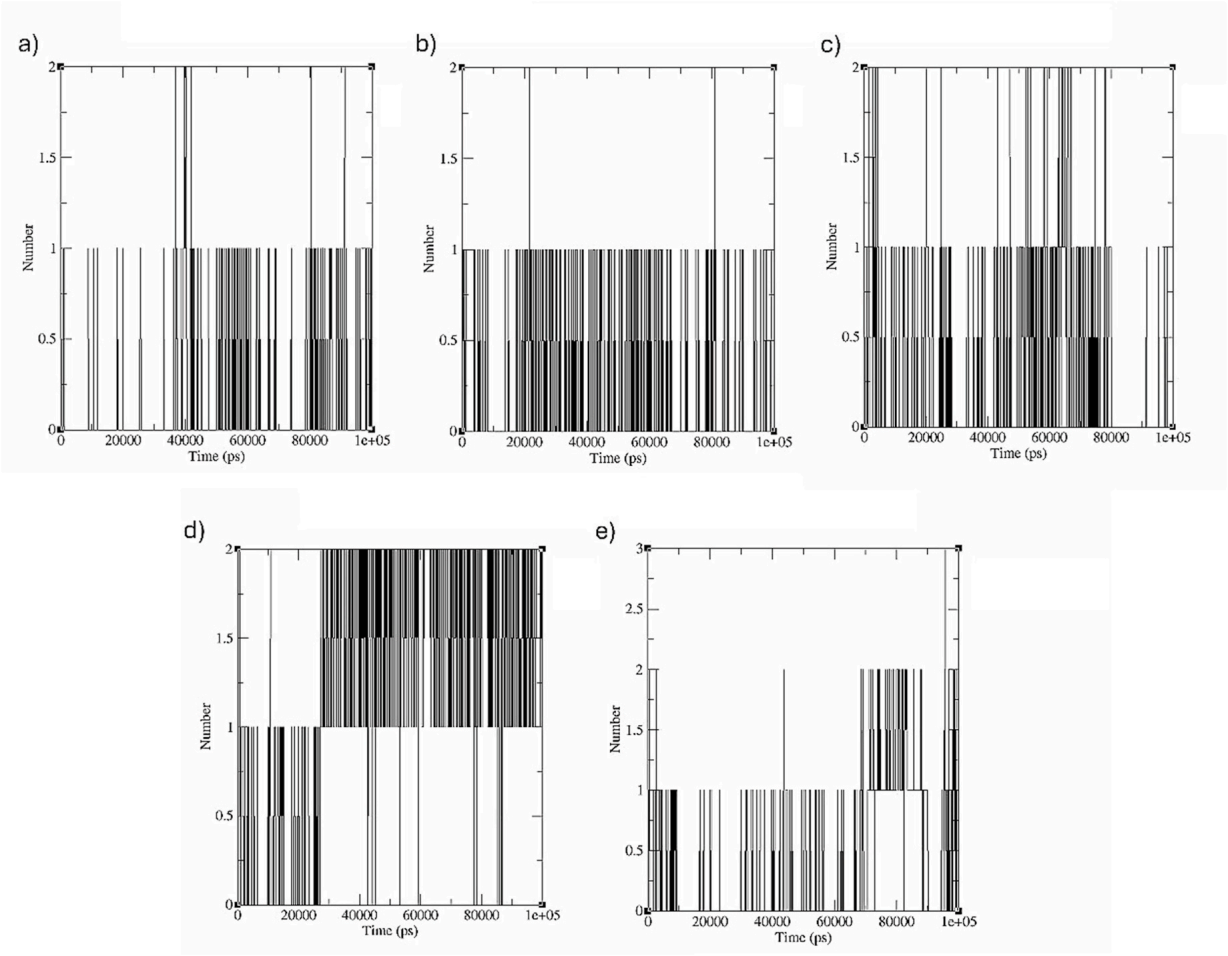
Hydrogen bond analysis for different ligands over 100 ns (a) epi-γ-eudesmol, (b) γ-eudesmol, (c) α-eudesmol, (d) α-cadinol and (e) epi-α-cadinol.

An investigation of hydrogen bonds (H-bonds) (Fig. 6a–e) was performed to assess the stability of the interactions within the binding pocket. Some ligands, such as epi-γ-eudesmol and γ-eudesmol, exhibit intermittent hydrogen bonding, with γ-eudesmol showing bond loss and reformation towards the end of the simulation. α-Eudesmol and epi-α-cadinol initially displayed some instability but retained H-bonds with Agr185 and Glu179 over time. Notably, epi-α-cadinol showed an increase in H-bond formation in later stages, suggesting progressive stabilization. Among all the ligands, α-cadinol exhibited the highest number of consistent hydrogen bonds throughout the simulation, indicating strong and stable binding with reduced ligand dissociation. Additionally, hydrophobic interactions with Leu183, Arg185, and Pro442, along with water-mediated van der Waals interactions, further contribute to complex stabilization.

**Fig. 7:**
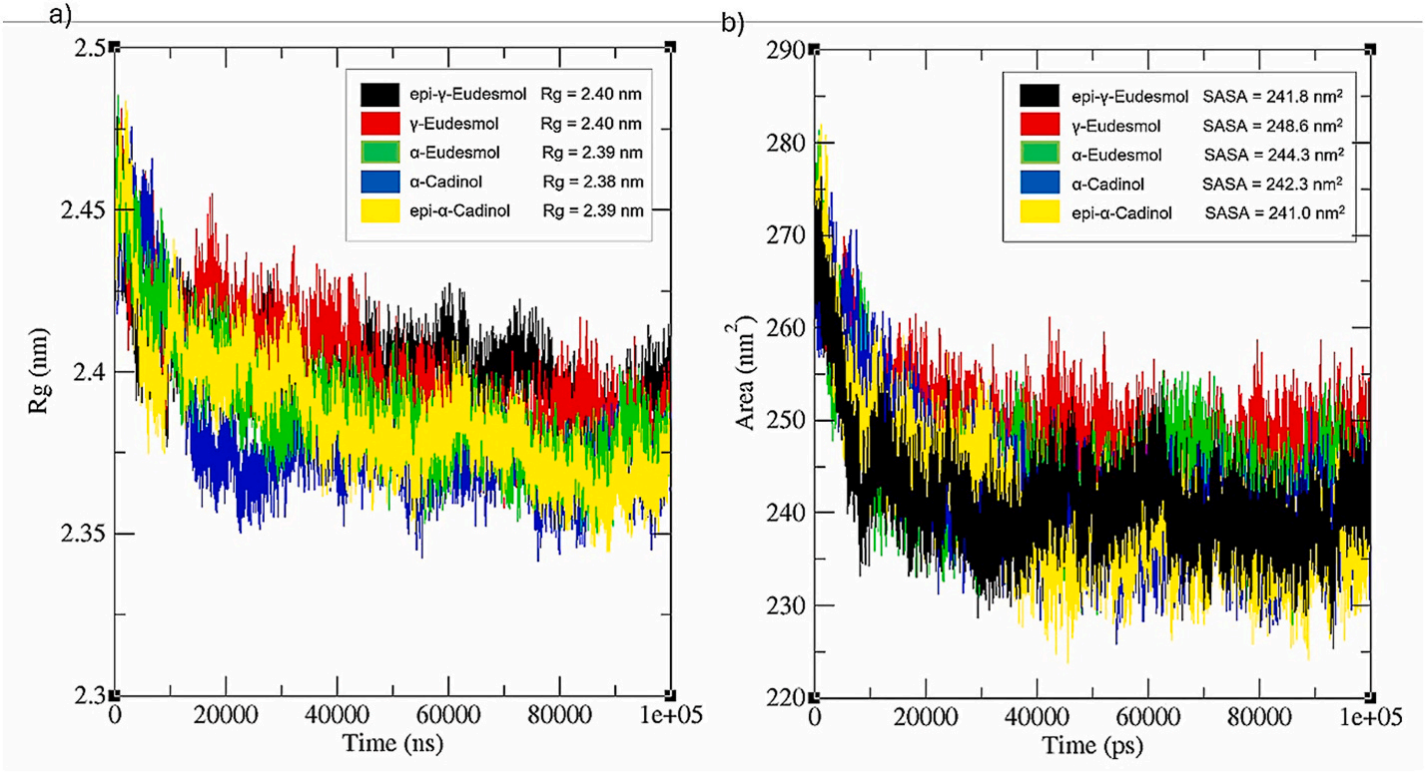
(a) Radius of Gyration (Rg) and (b) Solvent Accessible Surface Area (SASA) analysis of ligand-protein complexes.

The radius of gyration (Rg) plot (Fig. 7) revealed a general decline over time, indicating increased compactness of the protein-ligand complexes, suggesting structural stabilization. Although slight variations in the final Rg values were observed among the complexes, all exhibited similar levels of compactness upon ligand binding. Correspondingly, solvent-accessible surface area (SASA) studies indicated a reduction in solvent exposure with time, so reinforcing the notion that ligand binding enhances protein stability and folding. The absence of notable variations in SASA values across the ligands indicates that they exert a similar influence on protein stability.

The integrated assessment of RMSD, RMSF, hydrogen bonds, radius of gyration, and solvent-accessible surface area indicates that ligand binding is essential for the stabilization of the protein structure. Among the tested ligands, α-cadinol demonstrated the most stable binding because of its strong and persistent hydrogen bonding, whereas epi-α-cadinol exhibited gradual stabilization, adapting effectively to the protein's active site. These findings highlight the potential of α-cadinol and epi-α-cadinol as bioactive compounds for DOMS treatment.

## 4 Discussion

Patients with DOMS often receive anti-inflammatory drugs such as ibuprofen. Reports suggest that ibuprofen can alleviate the symptoms of DOMS, but does not aid in muscle repair. However, recent publications have found that ibuprofen is not as beneficial as a placebo [36,37]. Moreover, the use of anti-inflammatory drugs does not effectively prevent DOMS [38]. Treatment of DOMS requires alternative therapies.

This research examined the efficacy of *F. religiosa* in the management of delayed onset muscle soreness (DOMS). It employs the network pharmacology approach to construct and examine a network involving drugs, targets, and pathways. Molecular docking and molecular dynamics were employed to evaluate the binding affinity and dynamic behavior of prospective phytochemical substances.

Only 44 of the 140 chemical compounds from *F. religiosa* plants met the active drug candidate criteria for topical formulations. These compounds are predicted to be adsorbed onto the skin to act on receptors, causing the desired pharmacological effects.

The limitations of research and exploration of DOMS treatment targets compelled us to incorporate protein targets from the inflammatory pathway into the analysis to ensure the smooth implementation of network pharmacology. Indeed, the occurrence of DOMS is closely correlated with inflammation.

In this study, we predicted that *F. religiosa* compounds would bind to 39 DOMS protein targets. *F. religiosa* ‘s work on DOMS identified 10 pathways among these 39 targets as the primary pathways. Pathway enrichment analysis further identified the involvement of critical inflammatory and oxidative stress pathways, such as TNF and NF-kappa B signaling, which are pivotal in DOMS pathology.

Our study indicated that the PTGS2 protein, known as prostaglandin-endoperoxide synthase 2 (also termed prostaglandin G/H synthase and cyclooxygenase), was identified as the most promising protein target (Table 3). Cyclooxygenase, also known as prostaglandin-endoperoxide synthase, is the principal enzyme responsible for prostaglandin synthesis. It operates as both a dioxygenase and a peroxidase. PTGS consists of two isozymes: the constitutive PTGS1 and the inducible PTGS2, which demonstrate varying expression levels and tissue distribution. This gene encodes an inducible isoform of an enzyme. Particular stimulatory events govern this process, highlighting their significance in the production of prostanoid compounds linked to inflammation and mitogenesis.

We selected several *F. religiosa* compounds for docking with PTGS2 based on their potential as anti-inflammatory agents, role in DOMS pathology, and number of nodes in the network. The three compounds with the strongest bonds are α-eudesmol, epi-γ-eudesmol, and α-cadinol.

The leaves of *F. religiosa* plants contain these three compounds as essential oil components. Until now, there has been no testing of the effectiveness of these three compounds as analgesics, anti-inflammatory agents, or DOMS. However, studies have demonstrated the anti-inflammatory and analgesic properties of leaf extracts from the Bodhi plant [39]. Recent studies have shown that the leaves of *F. religiosa* plants also exhibit antioxidant activities [40]. High consumption of antioxidants is recommended to prevent and treat inflammation and muscle soreness [41]. Antioxidant therapy can treat oxidative stress caused by physical exercise [42]. Studying the use of *F. religiosa* in traditional polyherbal formulations for DOMS and examining its pharmacokinetic and pharmacodynamic profiles is also worth investigating.

The enhanced binding stability of the protein-ligand combination was validated using molecular dynamics analysis. Molecular dynamics simulations elucidate the dynamic interactions between pharmaceuticals and target molecules, offering comprehensive insights into their binding methods, stability, and interaction patterns [43]. These findings indicated that the PTGS2-α-cadinol complex exhibited robust and enduring hydrogen bonding. This aligns with the docking analysis findings. These data show that the PTGS2-α-cadinol and PTGS2-epi-α-cadinol complex may play a crucial role in the control of DOMS.

Alpha-cadinol and epi-α-cadinol are volatile compounds classified as sesquiterpenoids within the terpenoid group, and they have been identified in the essential oil of *F. religiosa* leaves [44]. No reports have indicated the presence of these compounds in any part of the plant other than the leaves, including the seeds [44]. Essential oil can be derived from seeds; however, they have primarily been documented to contain alkaloids, tannins, flavonoids, phenols, triterpenoids, cardiac glycosides, quinones, and coumarins [44,45].

Based on these findings, leaves may be considered a relevant plant part in this study, particularly due to the presence of identified volatile compounds. However, it is important to note that other plant parts, such as seeds, also contain bioactive constituents that may contribute to pharmacological effects. Therefore, the selection of leaves in this context is primarily supported by considerations of accessibility, renewability, and sustainable utilization, rather than implying phytochemical superiority over other plant parts.

## 5 Limitations

This study presents several limitations that must be acknowledged. The molecular docking and molecular dynamics simulations were initially conducted with a single receptor and a restricted selection of compounds, potentially failing to encompass the full range of molecular interactions. The findings are limited to an in silico framework; therefore, further validation through in vivo and clinical studies is necessary to confirm the predicted pharmacological effects and clarify the precise mechanisms of action.

The scope of this work is focused on providing pharmacological evidence for *F. religiosa* concerning musculoskeletal health, particularly its potential in alleviating muscle soreness, specifically DOMS. This focused strategy underscores the significance of *F. religiosa* as a potential subject for additional translational research, while acknowledging the necessity for thorough experimental and clinical studies.

## 6 Conclusion

This study explored the mechanisms by which *F. religiosa* can alleviate DOMS. It identified 44 bioavailable phytochemicals that interact with 39 potential therapeutic targets, including PTGS2, a key component of inflammatory pathways. Molecular docking studies showed that α-eudesmol, epi-γ-eudesmol, and α-cadinol have greater binding affinities to PTGS2 than to ibuprofen, suggesting potential anti-inflammatory and analgesic properties. This study also highlights the involvement of inflammatory and oxidative stress pathways, including TNF- and NF-kappa B signaling, in the pathogenesis of DOMS. Molecular dynamics simulations supported the stability of α-cadinol and epi-α-cadinol as bioactive molecules.

## Author contributions

Original idea from I Gusti Agung Ayu Kartika, Agung Endro Nugroho, Syazwani Itri Amran, and Sisca Ucche. Experimental design and data collection by I Gusti Agung Ayu Kartika, Kevin Awidarta, and Maysaa Alakbaree. Data analysis and confirmation by I Gusti Agung Ayu Kartika, Kevin Awidarta, Maysaa Alakbaree, Sisca Ucche, Syazwani Itri Amran, and Agung Endro Nugroho. Manuscript writing by I Gusti Agung Ayu Kartika, Kevin Awidarta, Maysaa Alakbaree, Sisca Ucche, Syazwani Itri Amran, and Agung Endro Nugroho. All authors have thoroughly revised the manuscript.

## Declaration of generative AI in scientific writing

The authors declare that this article does not use generative AI- or AI-assisted technology.

## Funding sources

This research received funding from the 10.13039/501100012521Universitas Gadjah Mada, Republic of Indonesia.

## Conflict of interest

The authors declare that they have no known competing financial interests or personal relationships that could have appeared to influence the work reported in this paper.
