# Supplementary material for: Deciphering the action mechanism of the sacred plant “Ashvattha” (Ficus religiosa L.) in the treatment of delayed-onset muscle soreness
Source: J Ayurveda Integr Med. 2026 Aug 5;17(5):101389. doi: 10.1016/j.jaim.2026.101389 (PMC13474386; doi:10.1016/j.jaim.2026.101389)
Supplement: Supplementary file 1 [file mmc1.docx]

**Fig. 1.** KEGG 2021 human

**Fig. 2.** The GO functional enrichment analysis: (A) Enriched Biological Process of potential target genes; (B) Enriched Cellular Component of potential target genes; and (C) Enriched Molecular Function of potential target genes

**Fig. 3.** KEGG pathway enrichment analysis. KEGG pathway name on Y-axis and rich factor on X-axis. The color from green to red reflects the adjusted log10 (P value) from large to small, and the size of the bubble indicates the number of related genes involved.

|  | |  |
| --- | --- | --- |
| **Native Ligand** | | |
|  |  | |
| **Ibuprofen** | | |
|  |  | |
| **α-Eudesmol** | | |
|  |  | |
| **epi-γ-Eudesmol** | | |
|  |  | |
| **epi-α-Cadinol** | | |
|  |  | |
| **α-Cadinol** | | |
|  |  | |
| **γ-Eudesmol** | | |

**Fig. 4.** The three-dimensional docking arrangements and two-dimensional ligand-protein interactions

**Table 1. Targets of *Ficus religiosa*, DOMS, and Their Overlapping**

| **No.** | **Targets of F. religiosa** | **Targets of DOMS** | **Overlapping Targets** |
| --- | --- | --- | --- |
| 1 | UGT2B7 | CMM | PTGER3 |
| 2 | AR | ISG15 | EIF2AK2 |
| 3 | JAK1 | TNFRSF4 | CNR2 |
| 4 | JAK2 | C1QTNF12 | PTPN22 |
| 5 | DRD2 | TNFRSF14 | GSTM1 |
| 6 | SIGMAR1 | TP73 | ELANE |
| 7 | HMOX1 | TNFRSF25 | CTRC |
| 8 | CYP11B1 | TNFRSF9 | FABP1 |
| 9 | CYP11B2 | PIK3CD | MB |
| 10 | JAK3 | MTOR | NQO1 |
| 11 | TYK2 | TNFRSF8 | DPP4 |
| 12 | IDO1 | TNFRSF1B | PLA2G2A |
| 13 | KCNH2 | PDPN | S1PR1 |
| 14 | NR3C2 | CTRC | ALK |
| 15 | PGR | PLA2G2E | ADORA1 |
| 16 | CA2 | PLA2G2A | CASP8 |
| 17 | CA1 | PLA2G2F | PTGFR |
| 18 | CA4 | PINK1 | SELE |
| 19 | EPHX2 | C1QB | NFE2L2 |
| 20 | CHRNA3 | CNR2 | MTOR |
| 21 | CHRNB4 | IL22RA1 | LCK |
| 22 | SLC6A3 | RUNX3 | CCR2 |
| 23 | SHBG | MATN1 | PLA2G4A |
| 24 | CYP19A1 | CCDC28B | ITGB6 |
| 25 | PIM1 | LCK | MPO |
| 26 | HSD17B2 | YARS1 | JAK1 |
| 27 | PRKCG | TRIM62 | ADAM17 |
| 28 | PRKCD | ZC3H12A | ITGAV |
| 29 | PRKCA | HPCAL4 | EGLN1 |
| 30 | PIM3 | MYCL | RORC |
| 31 | TRPV3 | CAP1 | PTGS2 |
| 32 | PER2 | GUCA2B | IL1B |
| 33 | PSEN2 | TIE1 | NCSTN |
| 34 | PSENEN | MUTYH | CTSK |
| 35 | NCSTN | RAD54L | PIK3CD |
| 36 | APH1A | TAL1 | JUN |
| 37 | PSEN1 | MYSM1 | SCN9A |
| 38 | APH1B | JUN | COMT |
| 39 | EPHX1 | JAK1 | PARP1 |
| 40 | CCNB3 | IL23R |  |
| 41 | CDK1 | IL12RB2 |  |
| 42 | CCNB1 | WLS |  |
| 43 | CCNB2 | PTGER3 |  |
| 44 | GSK3B | ASB17 |  |
| 45 | EPHA4 | PTGFR |  |
| 46 | KCNA3 | CCN1 |  |
| 47 | SCN5A | GFI1 |  |
| 48 | SCN9A | F3 |  |
| 49 | SQLE | S1PR1 |  |
| 50 | CDC25A | GSTM1 |  |
| 51 | POLA1 | CSF1 |  |
| 52 | MAPK3 | CHIA |  |
| 53 | AKR1C3 | RAP1A |  |
| 54 | GABRB3 | PTPN22 |  |
| 55 | GABRG2 | NRAS |  |
| 56 | GABRA5 | IGSF3 |  |
| 57 | CHRM4 | REG4 |  |
| 58 | TRPM8 | HJV |  |
| 59 | CHRM5 | FCGR1A |  |
| 60 | CHRM1 | CTSK |  |
| 61 | CHRM3 | SETDB1 |  |
| 62 | PRKCB | TNFAIP8L2 |  |
| 63 | CYP2C9 | SELENBP1 |  |
| 64 | PRKCE | PSMB4 |  |
| 65 | PRKCH | RORC |  |
| 66 | PRKCQ | FLG |  |
| 67 | MAP3K14 | FLG2 |  |
| 68 | RASGRP1 | SPRR2A |  |
| 69 | ADRA2C | SPRR2B |  |
| 70 | KCNMA1 | S100A9 |  |
| 71 | ADA | S100A12 |  |
| 72 | PARP1 | S100A8 |  |
| 73 | F2RL3 | IL6R |  |
| 74 | NPC1L1 | ADAR |  |
| 75 | SPHK2 | MUC1 |  |
| 76 | SPHK1 | GBA1 |  |
| 77 | RORA | LMNA |  |
| 78 | PYGL | TMEM79 |  |
| 79 | AURKA | GLMP |  |
| 80 | MELK | CD1D |  |
| 81 | ABCC9 | AIM2 |  |
| 82 | PTAFR | ACKR1 |  |
| 83 | HSD11B2 | FCER1A |  |
| 84 | HSD17B3 | CRP |  |
| 85 | HDAC2 | COPA |  |
| 86 | EGFR | NCSTN |  |
| 87 | ACPP | CD244 |  |
| 88 | SLC18A3 | NECTIN4 |  |
| 89 | CA5A | FCER1G |  |
| 90 | APP | FCGR2A |  |
| 91 | CHEK1 | FCGR3A |  |
| 92 | SLC6A2 | FCGR3B |  |
| 93 | LRRK2 | FCGR2B |  |
| 94 | TYMS | DDR2 |  |
| 95 | KCNE1 | F5 |  |
| 96 | KCNQ1 | SELP |  |
| 97 | CA6 | SELL |  |
| 98 | LTA4H | SELE |  |
| 99 | STK3 | FASLG |  |
| 100 | NOS1 | TNFSF4 |  |
| 101 | TBXAS1 | RC3H1 |  |
| 102 | ICMT | STX6 |  |
| 103 | CES1 | ARPC5 |  |
| 104 | AOC3 | HMCN1 |  |
| 105 | CA9 | PTGS2 |  |
| 106 | HMGCR | PLA2G4A |  |
| 107 | MPO | KCNT2 |  |
| 108 | PIK3CG | CFH |  |
| 109 | PIK3CA | CFHR3 |  |
| 110 | NPY5R | CFHR1 |  |
| 111 | BRD4 | NR5A2 |  |
| 112 | ALDH1A1 | ZNF281 |  |
| 113 | CHRM2 | ADIPOR1 |  |
| 114 | ADH1A | ADORA1 |  |
| 115 | ADH4 | CHI3L1 |  |
| 116 | ADH1B | CNTN2 |  |
| 117 | ADH1C | IKBKE |  |
| 118 | GLI2 | IL10 |  |
| 119 | GLI1 | IL24 |  |
| 120 | MAOA | CD55 |  |
| 121 | ADH7 | CR2 |  |
| 122 | CYP2A6 | CR1 |  |
| 123 | HSD11B1 | CD46 |  |
| 124 | MAOB | LAMB3 |  |
| 125 | CHRNA4 | TRAF5 |  |
| 126 | CHRNB2 | SLC30A10 |  |
| 127 | CYP2C19 | TLR5 |  |
| 128 | TRPA1 | PARP1 |  |
| 129 | NR4A1 | AGT |  |
| 130 | HSPA1A | EGLN1 |  |
| 131 | PTK2B | NLRP3 |  |
| 132 | DBF4 | TRIM58 |  |
| 133 | CDC7 | LYPD8 |  |
| 134 | ICAM1 | PXDN |  |
| 135 | SELE | CMPK2 |  |
| 136 | ALOX15 | ADAM17 |  |
| 137 | PSMB5 | SLC5A6 |  |
| 138 | KCNK2 | MPV17 |  |
| 139 | CCND1 | FNDC4 |  |
| 140 | CDK4 | ALK |  |
| 141 | AKR1B1 | NLRC4 |  |
| 142 | TTR | EIF2AK2 |  |
| 143 | PIM2 | SIX2 |  |
| 144 | MTNR1A | TTC7A |  |
| 145 | MTNR1B | EPCAM |  |
| 146 | TDP2 | CCDC88A |  |
| 147 | ESR1 | GKN2 |  |
| 148 | ESR2 | DYSF |  |
| 149 | CTSK | MTHFD2 |  |
| 150 | ABL1 | REG1A |  |
| 151 | NQO2 | VAMP8 |  |
| 152 | CA12 | CD8A |  |
| 153 | PTGS2 | FABP1 |  |
| 154 | NOTUM | CELIAC8 |  |
| 155 | TPO | COPD |  |
| 156 | TYMP | STARD7 |  |
| 157 | PARP2 | NEURL3 |  |
| 158 | CHRNA7 | MAP4K4 |  |
| 159 | NAAA | IL1R1 |  |
| 160 | IKBKB | IL1RL1 |  |
| 161 | FYN | BUB1 |  |
| 162 | GRM1 | MIR4435-2HG |  |
| 163 | PRF1 | MERTK |  |
| 164 | SLC22A12 | IL1A |  |
| 165 | IMPDH2 | IL1B |  |
| 166 | ROCK1 | IL37 |  |
| 167 | PRKACA | IL36G |  |
| 168 | RPS6KB1 | IL36A |  |
| 169 | HPGD | IL36B |  |
| 170 | HLCS | IL36RN |  |
| 171 | PTGS1 | IL1RN |  |
| 172 | HTR2C | MARCO |  |
| 173 | HSP90AA1 | PROC |  |
| 174 | ASAH1 | CXCR4 |  |
| 175 | RPS6KA3 | HNMT |  |
| 176 | TERT | NR4A2 |  |
| 177 | ALOX12 | GPD2 |  |
| 178 | PTPRC | ACVR1 |  |
| 179 | PTPN1 | ITGB6 |  |
| 180 | PTPRF | DPP4 |  |
| 181 | PTGDR2 | GCG |  |
| 182 | EPAS1 | IFIH1 |  |
| 183 | CETP | SCN1A |  |
| 184 | ADAMTS5 | SCN9A |  |
| 185 | GSR | G6PC2 |  |
| 186 | DUSP3 | NFE2L2 |  |
| 187 | AVPR2 | TTN |  |
| 188 | GRM5 | NEUROD1 |  |
| 189 | HSP90AB1 | ARVD4 |  |
| 190 | NOS2 | ITGAV |  |
| 191 | SRD5A1 | GULP1 |  |
| 192 | CASP3 | STAT1 |  |
| 193 | PARP10 | STAT4 |  |
| 194 | SIRT3 | C2orf69 |  |
| 195 | NOS3 | CASP10 |  |
| 196 | SLC5A7 | CASP8 |  |
| 197 | MGLL | CTLA4 |  |
| 198 | CA7 | ICOS |  |
| 199 | CA14 | IDDM13 |  |
| 200 | MCL1 | BARD1 |  |
| 201 | CYP3A4 | [COMT](https://www.genecards.org/cgi-bin/carddisp.pl?gene=COMT&keywords=Delayed%20Onset%20Muscle%20Soreness) |  |
| 202 | KAT2B | [MB](https://www.genecards.org/cgi-bin/carddisp.pl?gene=MB&keywords=Delayed%20Onset%20Muscle%20Soreness) |  |
| 203 | METAP1 | [SOD1](https://www.genecards.org/cgi-bin/carddisp.pl?gene=SOD1&keywords=Delayed%20Onset%20Muscle%20Soreness) |  |
| 204 | MAP2K1 | [DES](https://www.genecards.org/cgi-bin/carddisp.pl?gene=DES&keywords=Delayed%20Onset%20Muscle%20Soreness) |  |
| 205 | DUSP1 | [MPO](https://www.genecards.org/cgi-bin/carddisp.pl?gene=MPO&keywords=Delayed%20Onset%20Muscle%20Soreness) |  |
| 206 | HTR7 | [ELANE](https://www.genecards.org/cgi-bin/carddisp.pl?gene=ELANE&keywords=Delayed%20Onset%20Muscle%20Soreness) |  |
| 207 | DRD4 | [CAT](https://www.genecards.org/cgi-bin/carddisp.pl?gene=CAT&keywords=Delayed%20Onset%20Muscle%20Soreness) |  |
| 208 | SLC6A4 | [CCL2](https://www.genecards.org/cgi-bin/carddisp.pl?gene=CCL2&keywords=Delayed%20Onset%20Muscle%20Soreness) |  |
| 209 | TOP2A | [CHKA](https://www.genecards.org/cgi-bin/carddisp.pl?gene=CHKA&keywords=Delayed%20Onset%20Muscle%20Soreness) |  |
| 210 | PDE7A | [NQO1](https://www.genecards.org/cgi-bin/carddisp.pl?gene=NQO1&keywords=Delayed%20Onset%20Muscle%20Soreness) |  |
| 211 | NQO1 | [CCR2](https://www.genecards.org/cgi-bin/carddisp.pl?gene=CCR2&keywords=Delayed%20Onset%20Muscle%20Soreness) |  |
| 212 | PTPN2 | [GUSB](https://www.genecards.org/cgi-bin/carddisp.pl?gene=GUSB&keywords=Delayed%20Onset%20Muscle%20Soreness) |  |
| 213 | ACE | [MYH7](https://www.genecards.org/cgi-bin/carddisp.pl?gene=MYH7&keywords=Delayed%20Onset%20Muscle%20Soreness) |  |
| 214 | CASP1 | [ITGAM](https://www.genecards.org/cgi-bin/carddisp.pl?gene=ITGAM&keywords=Delayed%20Onset%20Muscle%20Soreness) |  |
| 215 | ACHE | [FLNC](https://www.genecards.org/cgi-bin/carddisp.pl?gene=FLNC&keywords=Delayed%20Onset%20Muscle%20Soreness) |  |
| 216 | PARP3 | [MYOT](https://www.genecards.org/cgi-bin/carddisp.pl?gene=MYOT&keywords=Delayed%20Onset%20Muscle%20Soreness) |  |
| 217 | BRD9 | [TRIM63](https://www.genecards.org/cgi-bin/carddisp.pl?gene=TRIM63&keywords=Delayed%20Onset%20Muscle%20Soreness) |  |
| 218 | PLK1 | [ACTN3](https://www.genecards.org/cgi-bin/carddisp.pl?gene=ACTN3&keywords=Delayed%20Onset%20Muscle%20Soreness) |  |
| 219 | P2RX7 | [TNNI1](https://www.genecards.org/cgi-bin/carddisp.pl?gene=TNNI1&keywords=Delayed%20Onset%20Muscle%20Soreness) |  |
| 220 | MALT1 | [OBSCN](https://www.genecards.org/cgi-bin/carddisp.pl?gene=OBSCN&keywords=Delayed%20Onset%20Muscle%20Soreness) |  |
| 221 | MIF | [MYOZ1](https://www.genecards.org/cgi-bin/carddisp.pl?gene=MYOZ1&keywords=Delayed%20Onset%20Muscle%20Soreness) |  |
| 222 | ALOX5 | [BDNF-AS](https://www.genecards.org/cgi-bin/carddisp.pl?gene=BDNF-AS&keywords=Delayed%20Onset%20Muscle%20Soreness) |  |
| 223 | SOAT1 | [SOD2-OT1](https://www.genecards.org/cgi-bin/carddisp.pl?gene=SOD2-OT1&keywords=Delayed%20Onset%20Muscle%20Soreness) |  |
| 224 | HPGDS | [IGF2-AS](https://www.genecards.org/cgi-bin/carddisp.pl?gene=IGF2-AS&keywords=Delayed%20Onset%20Muscle%20Soreness) |  |
| 225 | PTPN22 |  |  |
| 226 | HDAC8 |  |  |
| 227 | DNMT1 |  |  |
| 228 | TGM2 |  |  |
| 229 | DNMT3L |  |  |
| 230 | DNMT3A |  |  |
| 231 | PREP |  |  |
| 232 | RGS4 |  |  |
| 233 | CA3 |  |  |
| 234 | CA13 |  |  |
| 235 | CA5B |  |  |
| 236 | CES2 |  |  |
| 237 | MMP13 |  |  |
| 238 | MMP8 |  |  |
| 239 | TYR |  |  |
| 240 | HTR2B |  |  |
| 241 | CYP17A1 |  |  |
| 242 | HDAC4 |  |  |
| 243 | ADRA2A |  |  |
| 244 | ADRA2B |  |  |
| 245 | COMT |  |  |
| 246 | ALPL |  |  |
| 247 | SRD5A2 |  |  |
| 248 | DAO |  |  |
| 249 | MAP2 |  |  |
| 250 | IGF1R |  |  |
| 251 | ERN1 |  |  |
| 252 | FADS1 |  |  |
| 253 | HRH3 |  |  |
| 254 | HRH4 |  |  |
| 255 | VEGFA |  |  |
| 256 | EGLN1 |  |  |
| 257 | TLR9 |  |  |
| 258 | HDAC6 |  |  |
| 259 | METAP2 |  |  |
| 260 | MET |  |  |
| 261 | HDAC1 |  |  |
| 262 | CXCR2 |  |  |
| 263 | MMP9 |  |  |
| 264 | MMP1 |  |  |
| 265 | MMP2 |  |  |
| 266 | PLK4 |  |  |
| 267 | PLK2 |  |  |
| 268 | FGFR1 |  |  |
| 269 | DCTPP1 |  |  |
| 270 | TUBB1 |  |  |
| 271 | RNASEH1 |  |  |
| 272 | KDR |  |  |
| 273 | GPR84 |  |  |
| 274 | NISCH |  |  |
| 275 | AURKB |  |  |
| 276 | SRC |  |  |
| 277 | ROCK2 |  |  |
| 278 | FLT3 |  |  |
| 279 | CDK2 |  |  |
| 280 | CYP1A2 |  |  |
| 281 | CHUK |  |  |
| 282 | BRAF |  |  |
| 283 | PABPC1 |  |  |
| 284 | HTR6 |  |  |
| 285 | PDE10A |  |  |
| 286 | BCHE |  |  |
| 287 | THRB |  |  |
| 288 | TNKS2 |  |  |
| 289 | TSPO |  |  |
| 290 | PIK3CB |  |  |
| 291 | PDE5A |  |  |
| 292 | ABCB1 |  |  |
| 293 | HCAR2 |  |  |
| 294 | ELANE |  |  |
| 295 | PLA2G7 |  |  |
| 296 | QPCT |  |  |
| 297 | VCAM1 |  |  |
| 298 | CTSL |  |  |
| 299 | CTSB |  |  |
| 300 | C1R |  |  |
| 301 | PDE3A |  |  |
| 302 | CNR2 |  |  |
| 303 | PDE4A |  |  |
| 304 | PDE2A |  |  |
| 305 | PDE11A |  |  |
| 306 | PDE7B |  |  |
| 307 | GABRA3 |  |  |
| 308 | GABRA1 |  |  |
| 309 | GABRA2 |  |  |
| 310 | CTSH |  |  |
| 311 | XPO1 |  |  |
| 312 | CRHR1 |  |  |
| 313 | GABRB2 |  |  |
| 314 | CTSC |  |  |
| 315 | CTSF |  |  |
| 316 | CTSS |  |  |
| 317 | CTSV |  |  |
| 318 | RAPGEF4 |  |  |
| 319 | PIK3CD |  |  |
| 320 | PIK3R1 |  |  |
| 321 | HTR1A |  |  |
| 322 | GPBAR1 |  |  |
| 323 | GRM2 |  |  |
| 324 | CCR1 |  |  |
| 325 | CCR5 |  |  |
| 326 | CCR8 |  |  |
| 327 | ADORA3 |  |  |
| 328 | GPR55 |  |  |
| 329 | RORC |  |  |
| 330 | ADORA1 |  |  |
| 331 | GRM4 |  |  |
| 332 | RORB |  |  |
| 333 | VCP |  |  |
| 334 | ADORA2B |  |  |
| 335 | PRKDC |  |  |
| 336 | FNTA |  |  |
| 337 | FNTB |  |  |
| 338 | RELA |  |  |
| 339 | CDK5R1 |  |  |
| 340 | CDK5 |  |  |
| 341 | CYP51A1 |  |  |
| 342 | ALDH3A1 |  |  |
| 343 | ADRA1D |  |  |
| 344 | EDNRA |  |  |
| 345 | NFKBIA |  |  |
| 346 | PKM |  |  |
| 347 | KIF11 |  |  |
| 348 | EDNRB |  |  |
| 349 | CCNA1 |  |  |
| 350 | CCNA2 |  |  |
| 351 | CSNK2A1 |  |  |
| 352 | OXTR |  |  |
| 353 | PPARD |  |  |
| 354 | SLC16A1 |  |  |
| 355 | BRD2 |  |  |
| 356 | FBP1 |  |  |
| 357 | CYP2D6 |  |  |
| 358 | PFKFB4 |  |  |
| 359 | PFKFB3 |  |  |
| 360 | OPRK1 |  |  |
| 361 | KDM1A |  |  |
| 362 | FKBP1A |  |  |
| 363 | FDFT1 |  |  |
| 364 | AHCY |  |  |
| 365 | CCR2 |  |  |
| 366 | GRK2 |  |  |
| 367 | TLR4 |  |  |
| 368 | KMO |  |  |
| 369 | ADAM17 |  |  |
| 370 | NR3C1 |  |  |
| 371 | FAAH |  |  |
| 372 | KCNJ11 |  |  |
| 373 | BDKRB1 |  |  |
| 374 | FLT1 |  |  |
| 375 | KIT |  |  |
| 376 | EIF2AK3 |  |  |
| 377 | TAAR1 |  |  |
| 378 | LIPA |  |  |
| 379 | P2RX3 |  |  |
| 380 | GLRA2 |  |  |
| 381 | SLC5A1 |  |  |
| 382 | KDM5C |  |  |
| 383 | MAPK14 |  |  |
| 384 | KDM4B |  |  |
| 385 | KDM5B |  |  |
| 386 | KDM4A |  |  |
| 387 | KCNE1 |  |  |
| 388 | RXRB |  |  |
| 389 | RARG |  |  |
| 390 | RXRG |  |  |
| 391 | RARB |  |  |
| 392 | RARA |  |  |
| 393 | RXRA |  |  |
| 394 | MAPK1 |  |  |
| 395 | MPI |  |  |
| 396 | HAO1 |  |  |
| 397 | SIRT2 |  |  |
| 398 | TDO2 |  |  |
| 399 | AHR |  |  |
| 400 | BACE1 |  |  |
| 401 | SERPINA6 |  |  |
| 402 | NR1I2 |  |  |
| 403 | FABP1 |  |  |
| 404 | RASGRP3 |  |  |
| 405 | AKT1 |  |  |
| 406 | RBP4 |  |  |
| 407 | NR1I3 |  |  |
| 408 | PIN1 |  |  |
| 409 | CACNA1B |  |  |
| 410 | ADORA2A |  |  |
| 411 | KCNJ1 |  |  |
| 412 | SAE1 |  |  |
| 413 | ADRA1B |  |  |
| 414 | F2 |  |  |
| 415 | TLR7 |  |  |
| 416 | GPR35 |  |  |
| 417 | PLAU |  |  |
| 418 | ERBB2 |  |  |
| 419 | GSK3A |  |  |
| 420 | OPRD1 |  |  |
| 421 | MAPK8 |  |  |
| 422 | MAPK9 |  |  |
| 423 | TGFBR1 |  |  |
| 424 | CDK9 |  |  |
| 425 | CCNT1 |  |  |
| 426 | MKNK1 |  |  |
| 427 | PDPK1 |  |  |
| 428 | CCR4 |  |  |
| 429 | CCNE2 |  |  |
| 430 | CCNE1 |  |  |
| 431 | PRKCZ |  |  |
| 432 | SIRT1 |  |  |
| 433 | ALDH5A1 |  |  |
| 434 | ABAT |  |  |
| 435 | MME |  |  |
| 436 | REN |  |  |
| 437 | PLA2G5 |  |  |
| 438 | LAP3 |  |  |
| 439 | ACE2 |  |  |
| 440 | FOLH1 |  |  |
| 441 | CPA3 |  |  |
| 442 | ITGA2B |  |  |
| 443 | ITGB3 |  |  |
| 444 | KDM6B |  |  |
| 445 | EGLN3 |  |  |
| 446 | RNPEP |  |  |
| 447 | MMP12 |  |  |
| 448 | KDM4E |  |  |
| 449 | SLCO1B1 |  |  |
| 450 | KDM4D |  |  |
| 451 | PLA2G2A |  |  |
| 452 | PTGDR |  |  |
| 453 | HLA-A |  |  |
| 454 | DNPEP |  |  |
| 455 | PLA2G2C |  |  |
| 456 | PLA2G10 |  |  |
| 457 | PDE9A |  |  |
| 458 | CNR1 |  |  |
| 459 | PPP1CC |  |  |
| 460 | PLA2G4A |  |  |
| 461 | PPM1B |  |  |
| 462 | PARP15 |  |  |
| 463 | SORT1 |  |  |
| 464 | CAPN1 |  |  |
| 465 | GNPAT |  |  |
| 466 | PAM |  |  |
| 467 | KDM3A |  |  |
| 468 | CTSD |  |  |
| 469 | TAS1R3 |  |  |
| 470 | TAS1R1 |  |  |
| 471 | CSNK2A2 |  |  |
| 472 | HCAR3 |  |  |
| 473 | PPARG |  |  |
| 474 | DPP4 |  |  |
| 475 | XIAP |  |  |
| 476 | FPR2 |  |  |
| 477 | PGC |  |  |
| 478 | CXCL8 |  |  |
| 479 | ABCC1 |  |  |
| 480 | NR1H4 |  |  |
| 481 | ITGAV |  |  |
| 482 | CXCR1 |  |  |
| 483 | AKR1B10 |  |  |
| 484 | PPARA |  |  |
| 485 | AKR1C2 |  |  |
| 486 | AKR1A1 |  |  |
| 487 | HRH1 |  |  |
| 488 | EEF2K |  |  |
| 489 | FKBP5 |  |  |
| 490 | NR1D1 |  |  |
| 491 | HSD17B1 |  |  |
| 492 | ALB |  |  |
| 493 | LDHA |  |  |
| 494 | LDHB |  |  |
| 495 | DYRK1A |  |  |
| 496 | CLK1 |  |  |
| 497 | DYRK1B |  |  |
| 498 | FUT7 |  |  |
| 499 | FASN |  |  |
| 500 | DRD3 |  |  |
| 501 | CDC25B |  |  |
| 502 | AKR1C1 |  |  |
| 503 | ALK |  |  |
| 504 | DBH |  |  |
| 505 | MB |  |  |
| 506 | BCL2L1 |  |  |
| 507 | EP300 |  |  |
| 508 | ALPG |  |  |
| 509 | PLAA |  |  |
| 510 | STS |  |  |
| 511 | BCL2 |  |  |
| 512 | KCNA1 |  |  |
| 513 | KCNA4 |  |  |
| 514 | CFTR |  |  |
| 515 | NAT1 |  |  |
| 516 | MPG |  |  |
| 517 | HDAC3 |  |  |
| 518 | SERPINE1 |  |  |
| 519 | GSTA1 |  |  |
| 520 | POLB |  |  |
| 521 | KMT5A |  |  |
| 522 | PDE4B |  |  |
| 523 | PDE4D |  |  |
| 524 | HTR1E |  |  |
| 525 | ADRA1A |  |  |
| 526 | OPRM1 |  |  |
| 527 | MGMT |  |  |
| 528 | CISD1 |  |  |
| 529 | STAT3 |  |  |
| 530 | MAPK10 |  |  |
| 531 | HTR5A |  |  |
| 532 | DYRK2 |  |  |
| 533 | F12 |  |  |
| 534 | HDAC5 |  |  |
| 535 | NFKB1 |  |  |
| 536 | XDH |  |  |
| 537 | NUDT1 |  |  |
| 538 | LIMK1 |  |  |
| 539 | CTSA |  |  |
| 540 | CTRC |  |  |
| 541 | PDGFRB |  |  |
| 542 | FLT4 |  |  |
| 543 | INSR |  |  |
| 544 | PTK2 |  |  |
| 545 | TEK |  |  |
| 546 | MAP3K8 |  |  |
| 547 | EPHB4 |  |  |
| 548 | NUAK1 |  |  |
| 549 | FGR |  |  |
| 550 | LYN |  |  |
| 551 | HTT |  |  |
| 552 | F13A1 |  |  |
| 553 | CTRB1 |  |  |
| 554 | CYP1B1 |  |  |
| 555 | KDM5A |  |  |
| 556 | SLC22A6 |  |  |
| 557 | KCNN4 |  |  |
| 558 | CACNA2D1 |  |  |
| 559 | EWS-Fli1 |  |  |
| 560 | HTR1D |  |  |
| 561 | DHODH |  |  |
| 562 | PLAUR |  |  |
| 563 | ALDH2 |  |  |
| 564 | TNNC1 |  |  |
| 565 | TNNT2 |  |  |
| 566 | TNNI3 |  |  |
| 567 | CBR1 |  |  |
| 568 | CASP6 |  |  |
| 569 | CASP7 |  |  |
| 570 | CASP8 |  |  |
| 571 | CASP2 |  |  |
| 572 | APEX1 |  |  |
| 573 | KCNA5 |  |  |
| 574 | AXL |  |  |
| 575 | LCK |  |  |
| 576 | PON1 |  |  |
| 577 | GRK6 |  |  |
| 578 | PCSK7 |  |  |
| 579 | DDAH1 |  |  |
| 580 | UPP1 |  |  |
| 581 | SLC16A3 |  |  |
| 582 | PRSS1 |  |  |
| 583 | PLA2G6 |  |  |
| 584 | TNKS |  |  |
| 585 | PSMB1 |  |  |
| 586 | CTSG |  |  |
| 587 | GLP1R |  |  |
| 588 | LIPE |  |  |
| 589 | DHFR |  |  |
| 590 | CACNA1C |  |  |
| 591 | PLG |  |  |
| 592 | KLK1 |  |  |
| 593 | C1S |  |  |
| 594 | PBRM1 |  |  |
| 595 | CBFB |  |  |
| 596 | PGGT1B |  |  |
| 597 | CCND3 |  |  |
| 598 | CDK6 |  |  |
| 599 | FABP4 |  |  |
| 600 | FABP3 |  |  |
| 601 | TPMT |  |  |
| 602 | PLA2G2D |  |  |
| 603 | WDR5 |  |  |
| 604 | SLC7A11 |  |  |
| 605 | PLEC |  |  |
| 606 | ANPEP |  |  |
| 607 | ENPEP |  |  |
| 608 | PHLPP2 |  |  |
| 609 | F10 |  |  |
| 610 | CMA1 |  |  |
| 611 | EIF2AK2 |  |  |
| 612 | PTGER1 |  |  |
| 613 | PTGER4 |  |  |
| 614 | PTGFR |  |  |
| 615 | PTGER3 |  |  |
| 616 | PTGER2 |  |  |
| 617 | PTGIR |  |  |
| 618 | LTB4R |  |  |
| 619 | FABP5 |  |  |
| 620 | G6PD |  |  |
| 621 | GCK |  |  |
| 622 | MMP3 |  |  |
| 623 | THRA |  |  |
| 624 | GCGR |  |  |
| 625 | GIPR |  |  |
| 626 | ADRB2 |  |  |
| 627 | ADRB1 |  |  |
| 628 | ADRB3 |  |  |
| 629 | SSTR5 |  |  |
| 630 | DGAT1 |  |  |
| 631 | HTR2A |  |  |
| 632 | CD81 |  |  |
| 633 | TYRO3 |  |  |
| 634 | DPP7 |  |  |
| 635 | TBXA2R |  |  |
| 636 | MAP2K7 |  |  |
| 637 | GART |  |  |
| 638 | CPT1A |  |  |
| 639 | LIPC |  |  |
| 640 | CPT1B |  |  |
| 641 | LIPG |  |  |
| 642 | CASR |  |  |
| 643 | TOP1 |  |  |
| 644 | S1PR1 |  |  |
| 645 | GRB2 |  |  |
| 646 | TNF |  |  |
| 647 | PLA2G4B |  |  |
| 648 | KEAP1 |  |  |
| 649 | ITGB5 |  |  |
| 650 | ITGB6 |  |  |
| 651 | SCN4A |  |  |
| 652 | SCN2A |  |  |
| 653 | HDAC7 |  |  |
| 654 | EBP |  |  |
| 655 | ALOX15B |  |  |
| 656 | PTGES |  |  |
| 657 | DRD5 |  |  |
| 658 | DRD1 |  |  |
| 659 | HTR3A |  |  |
| 660 | HTR1B |  |  |
| 661 | GABBR2 |  |  |
| 662 | GABBR1 |  |  |
| 663 | LSS |  |  |
| 664 | VDR |  |  |
| 665 | ATP12A |  |  |
| 666 | RPS6KA5 |  |  |
| 667 | TAS2R31 |  |  |
| 668 | PDCD4 |  |  |
| 669 | TTL |  |  |
| 670 | PLA2G1B |  |  |
| 671 | CDC25C |  |  |
| 672 | PPP2CA |  |  |
| 673 | IARS |  |  |
| 674 | IL1B |  |  |
| 675 | F2RL1 |  |  |
| 676 | PTPN11 |  |  |
| 677 | ATP1A1 |  |  |
| 678 | JUN |  |  |
| 679 | ACP1 |  |  |
| 680 | HIF1A |  |  |
| 681 | GSTM1 |  |  |
| 682 | ITGAL |  |  |
| 683 | EHMT2 |  |  |
| 684 | TK1 |  |  |
| 685 | HPRT1 |  |  |
| 686 | TRPV1 |  |  |
| 687 | BBOX1 |  |  |
| 688 | ADK |  |  |
| 689 | CD38 |  |  |
| 690 | CLK4 |  |  |
| 691 | CLK2 |  |  |
| 692 | TMIGD3 |  |  |
| 693 | BCAT2 |  |  |
| 694 | SERPINH1 |  |  |
| 695 | FTO |  |  |
| 696 | PNP |  |  |
| 697 | STK26 |  |  |
| 698 | UTS2R |  |  |
| 699 | EIF2AK1 |  |  |
| 700 | MDM2 |  |  |
| 701 | HCRTR2 |  |  |
| 702 | HCRTR1 |  |  |
| 703 | BRD3 |  |  |
| 704 | SHH |  |  |
| 705 | NR1H3 |  |  |
| 706 | FFAR1 |  |  |
| 707 | FABP2 |  |  |
| 708 | PAOX |  |  |
| 709 | CEL |  |  |
| 710 | HASPIN |  |  |
| 711 | PHF8 |  |  |
| 712 | KDM2A |  |  |
| 713 | GSTK1 |  |  |
| 714 | UBA2 |  |  |
| 715 | CDC45 |  |  |
| 716 | SLC6A12 |  |  |
| 717 | PEPD |  |  |
| 718 | ITGB2 |  |  |
| 719 | GRIK1 |  |  |
| 720 | GRIK2 |  |  |
| 721 | SCD |  |  |
| 722 | PTPN6 |  |  |
| 723 | SLC1A2 |  |  |
| 724 | PTPRG |  |  |
| 725 | RPA1 |  |  |
| 726 | PSMB8 |  |  |
| 727 | HDAC9 |  |  |
| 728 | PDE3B |  |  |
| 729 | CYP1A1 |  |  |
| 730 | TUBB3 |  |  |
| 731 | NFE2L2 |  |  |
| 732 | MAPT |  |  |
| 733 | SYK |  |  |
| 734 | NPR1 |  |  |
| 735 | RET |  |  |
| 736 | SNCA |  |  |
| 737 | TLR8 |  |  |
| 738 | CXCR3 |  |  |
| 739 | ALOX5AP |  |  |
| 740 | CCR9 |  |  |
| 741 | AGTR2 |  |  |
| 742 | AGTR1 |  |  |
| 743 | ENPP2 |  |  |
| 744 | CCKBR |  |  |
| 745 | KDM2B |  |  |
| 746 | FFAR4 |  |  |
| 747 | ECE1 |  |  |
| 748 | HNF4A |  |  |
| 749 | MARS |  |  |
| 750 | AKR1C4 |  |  |
| 751 | ITGB1 |  |  |
| 752 | ITGA2 |  |  |
| 753 | HDAC10 |  |  |
| 754 | ADAMTS4 |  |  |
| 755 | AMPD3 |  |  |
| 756 | CTNNB1 |  |  |
| 757 | F2R |  |  |
| 758 | S1PR3 |  |  |
| 759 | AVPR1A |  |  |
| 760 | HSD3B1 |  |  |
| 761 | PLAT |  |  |
| 762 | ACACB |  |  |
| 763 | ABHD6 |  |  |
| 764 | MTOR |  |  |
| 765 | PLK3 |  |  |
| 766 | PIK3C2B |  |  |
| 767 | SREBF2 |  |  |
| 768 | SLC10A2 |  |  |
| 769 | NR1H2 |  |  |
| 770 | DHCR7 |  |  |
| 771 | GLRA1 |  |  |
| 772 | FNTA FNTB |  |  |
| 773 | CDK8 |  |  |
| 774 | C5AR1 |  |  |
| 775 | IL6ST |  |  |
| 776 | GC |  |  |
| 777 | MSR1 |  |  |
| 778 | EZH2 |  |  |

**Table 2. Name of Compounds and Their Pure Chemical Compounds Inventory Database (PCID)**

| **Compound No.** | **Compound Name** | **PCID** |
| --- | --- | --- |
| 1 | (2E)-Nonen-1-ol | 5364941 |
| 2 | (2E,6Z)-Nonadienal | 643731 |
| 3 | (2Z)-Hexenal | 6428782 |
| 4 | (3Z)-Hexenol | 10057450 |
| 5 | (3Z)-Hexenyl-tiglate | 15461361 |
| 6 | (E)-Cinnamyl alcohol | 5315892 |
| 7 | (E)-Linalool-oxide | 6432254 |
| 8 | (E)-β-Ionone | 348291614 |
| 9 | 1H-Benzopyran-1-one,3,4-dihydro-8-hydroxy-3-methyl | 28516 |
| 10 | 2-(Acetylamino)hexanoic acid | 306142 |
| 11 | 2,4-Decadienal(E,E) | 5283349 |
| 12 | 2,5-Dihydroxybenzaldehyde | 70949 |
| 13 | 2,6-Dimethoxyphenol | 7041 |
| 14 | 2-Phenylethyl-alcohol | 6054 |
| 15 | 4-Hydroxycoumarin | 54682930 |
| 16 | Allyl-caproate | 31266 |
| 17 | Benzoic-acid | 243 |
| 18 | Benzyl alcohol | 244 |
| 19 | Corchorifatty-acid-F | 348291029 |
| 20 | Coumaran | 10329 |
| 21 | Dihydroactinidiolide | 27209 |
| 22 | Ethanol, 2-phenoxy | 31236 |
| 23 | Eugenol | 3314 |
| 24 | Linalool | 6549 |
| 25 | n-Hexanol | 8103 |
| 26 | n-Nonanal | 31289 |
| 27 | Nonanoic-acid | 8158 |
| 28 | Octanoic-acid | 379 |
| 29 | Phenol | 996 |
| 30 | Phenylacetaldehyde | 998 |
| 31 | p-Vinylguaiacol | 332 |
| 32 | (3Z)-Hexenyl-benzoate | 101687121 |
| 33 | (E)-β-ocimene | 348285468 |
| 34 | 1,2-Benzenedicarboxylic-acid,mono(2-ethylhexyl)ester | 20393 |
| 35 | dendrolasine | 5316534 |
| 36 | epi-α-Cadinol | 348291693 |
| 37 | epi-γ-Eudesmol | 6428429 |
| 38 | limonene | 22311 |
| 39 | α-Cadinol | 317229097 |
| 40 | α-Copaene-11-ol | 249925971 |
| 41 | α-Eudesmol | 482043036 |
| 42 | α-terpinene | 481106870 |
| 43 | β-Eudesmol | 482040418 |
| 44 | γ-Eudesmol | 482032650 |

**Table 3. PCID of Compounds and Their Targets**

| **Compound No.** | **PCID** | **Target** |
| --- | --- | --- |
| 1 | 5364941 | JAK1 |
|  | 5364941 | NCSTN |
|  | 5364941 | SCN9A |
|  | 5364941 | PARP1 |
|  | 5364941 | MPO |
| 2 | 643731 | SELE |
|  | 643731 | JAK1 |
|  | 643731 | PARP1 |
|  | 643731 | CTSK |
|  | 643731 | PTGS2 |
|  | 643731 | MPO |
| 3 | 6428782 | CTSK |
|  | 6428782 | PARP1 |
|  | 6428782 | MPO |
|  | 6428782 | NQO1 |
|  | 6428782 | PTGS2 |
|  | 6428782 | PTPN22 |
| 4 | 10057450 | COMT |
|  | 10057450 | PTGS2 |
|  | 10057450 | EGLN1 |
|  | 10057450 | PARP1 |
|  | 10057450 | PTPN22 |
| 5 | 15461361 | CTSK |
|  | 15461361 | ELANE |
|  | 15461361 | SELE |
|  | 15461361 | CNR2 |
|  | 15461361 | PIK3CD |
|  | 15461361 | RORC |
|  | 15461361 | ADORA1 |
| 6 | 5315892 | JAK1 |
|  | 5315892 | MPO |
|  | 5315892 | PARP1 |
|  | 5315892 | NCSTN |
|  | 5315892 | CCR2 |
|  | 5315892 | COMT |
|  | 5315892 | ADAM17 |
| 7 | 6432254 | JAK1 |
|  | 6432254 | NCSTN |
|  | 6432254 | MPO |
|  | 6432254 | PTGS2 |
|  | 6432254 | PARP1 |
|  | 6432254 | PIK3CD |
| 8 | 348291614 | RORC |
|  | 348291614 | MPO |
|  | 348291614 | PARP1 |
|  | 348291614 | CTSK |
|  | 348291614 | FABP1 |
|  | 348291614 | NCSTN |
|  | 348291614 | PTGS2 |
| 9 | 28516 | ADORA1 |
|  | 28516 | EGLN1 |
|  | 28516 | COMT |
|  | 28516 | PARP1 |
|  | 28516 | JAK1 |
|  | 28516 | CCR2 |
| 10 | 306142 | PTGS2 |
|  | 306142 | PLA2G2A |
|  | 306142 | PLA2G4A |
|  | 306142 | PARP1 |
|  | 306142 | DPP4 |
|  | 306142 | CNR2 |
|  | 306142 | ITGAV |
|  | 306142 | FABP1 |
| 11 | 5283349 | RORC |
|  | 5283349 | PTGS2 |
|  | 5283349 | SELE |
|  | 5283349 | PARP1 |
|  | 5283349 | MPO |
|  | 5283349 | JAK1 |
|  | 5283349 | CTSK |
|  | 5283349 | CCR2 |
| 12 | 70949 | COMT |
|  | 70949 | PARP1 |
|  | 70949 | MPO |
|  | 70949 | ALK |
|  | 70949 | MB |
|  | 70949 | PTPN22 |
|  | 70949 | PIK3CD |
|  | 70949 | DPP4 |
|  | 70949 | ELANE |
|  | 70949 | CNR2 |
| 13 | 7041 | PARP1 |
|  | 7041 | MB |
|  | 7041 | NQO1 |
|  | 7041 | ELANE |
| 14 | 6054 | NQO1 |
|  | 6054 | CTRC |
|  | 6054 | CTSK |
|  | 6054 | ADORA1 |
|  | 6054 | PTGS2 |
| 15 | 54682930 | MPO |
|  | 54682930 | PARP1 |
|  | 54682930 | PTGS2 |
|  | 54682930 | JAK1 |
|  | 54682930 | NCSTN |
|  | 54682930 | PIK3CD |
|  | 54682930 | PTGS2 |
|  | 54682930 | NQO1 |
|  | 54682930 | CASP8 |
|  | 54682930 | ALK |
|  | 54682930 | CNR2 |
|  | 54682930 | LCK |
| 16 | 31266 | CTRC |
|  | 31266 | ELANE |
|  | 31266 | CTSK |
|  | 31266 | PIK3CD |
|  | 31266 | ADORA1 |
|  | 31266 | MPO |
|  | 31266 | SELE |
| 17 | 243 | PARP1 |
|  | 243 | DPP4 |
|  | 243 | COMT |
|  | 243 | ELANE |
|  | 243 | PTGS2 |
| 18 | 244 | MPO |
|  | 244 | PARP1 |
|  | 244 | ADORA1 |
|  | 244 | SCN9A |
|  | 244 | PIK3CD |
|  | 244 | EIF2AK2 |
| 19 | 348291029 | PTGFR |
|  | 348291029 | PTGER3 |
|  | 348291029 | FABP1 |
|  | 348291029 | LCK |
|  | 348291029 | DPP4 |
|  | 348291029 | S1PR1 |
|  | 348291029 | ADORA1 |
|  | 348291029 | ITGAV |
|  | 348291029 | ITGB6 |
| 20 | 10329 | CNR2 |
|  | 10329 | PTGS2 |
|  | 10329 | CTRC |
|  | 10329 | MPO |
| 21 | 27209 | PLA2G2A |
|  | 27209 | IL1B |
|  | 27209 | PTGS2 |
|  | 27209 | RORC |
|  | 27209 | CTSK |
|  | 27209 | JUN |
|  | 27209 | PARP1 |
|  | 27209 | GSTM1 |
|  | 27209 | MPO |
|  | 27209 | ADORA1 |
| 22 | 31236 | NQO1 |
|  | 31236 | MPO |
|  | 31236 | COMT |
|  | 31236 | ADORA1 |
|  | 31236 | ADAM17 |
|  | 31236 | PARP1 |
|  | 31236 | JAK1 |
|  | 31236 | SCN9A |
| 23 | 3314 | EGLN1 |
|  | 3314 | PARP1 |
|  | 3314 | ADORA1 |
|  | 3314 | PTPN22 |
|  | 3314 | JAK1 |
|  | 3314 | MB |
| 24 | 6549 | JAK1 |
|  | 6549 | PTGS2 |
|  | 6549 | SCN9A |
|  | 6549 | PARP1 |
|  | 6549 | NCSTN |
|  | 6549 | ADORA1 |
| 25 | 8103 | DPP4 |
|  | 8103 | JAK1 |
|  | 8103 | PARP1 |
|  | 8103 | PTGS2 |
|  | 8103 | MPO |
| 26 | 31289 | JAK1 |
|  | 31289 | CNR2 |
|  | 31289 | SELE |
|  | 31289 | PARP1 |
| 27 | 8158 | PTGFR |
|  | 8158 | PTGS2 |
|  | 8158 | PTGER3 |
|  | 8158 | FABP1 |
|  | 8158 | EGLN1 |
| 28 | 379 | PTGFR |
|  | 379 | PTGS2 |
|  | 379 | PTGER3 |
|  | 379 | CCR2 |
|  | 379 | EGLN1 |
| 29 | 996 | PTPN22 |
|  | 996 | PARP1 |
|  | 996 | NQO1 |
| 30 | 998 | PARP1 |
|  | 998 | CTSK |
|  | 998 | MPO |
|  | 998 | PTPN22 |
|  | 998 | PTGS2 |
| 31 | 332 | PARP1 |
|  | 332 | EGLN1 |
|  | 332 | NFE2L2 |
|  | 332 | PTGS2 |
|  | 332 | ADORA1 |
|  | 332 | JAK1 |
| 32 | 101687121 | ELANE |
|  | 101687121 | PTGS2 |
|  | 101687121 | SELE |
|  | 101687121 | CTSK |
|  | 101687121 | PIK3CD |
|  | 101687121 | CNR2 |
|  | 101687121 | ALK |
|  | 101687121 | PLA2G2A |
| 33 | 348285468 | CNR2 |
| 34 | 20393 | PTGS2 |
|  | 20393 | CCR2 |
|  | 20393 | FABP1 |
|  | 20393 | PTGER3 |
|  | 20393 | PTPN22 |
|  | 20393 | ITGAV |
| 35 | 5316534 | PTGS2 |
|  | 5316534 | SCN9A |
|  | 5316534 | PIK3CD |
|  | 5316534 | S1PR1 |
|  | 5316534 | MTOR |
| 36 | 348291693 | RORC |
|  | 348291693 | FABP1 |
|  | 348291693 | PTGS2 |
|  | 348291693 | NCSTN |
|  | 348291693 | JAK1 |
|  | 348291693 | PTPN22 |
| 37 | 6428429 | RORC |
|  | 6428429 | FABP1 |
|  | 6428429 | PTGS2 |
|  | 6428429 | NCSTN |
|  | 6428429 | JAK1 |
|  | 6428429 | PTPN22 |
|  | 6428429 | CNR2 |
| 38 | 22311 | CNR2 |
|  | 22311 | ADORA1 |
|  | 22311 | RORC |
|  | 22311 | FABP1 |
| 39 | 317229097 | RORC |
|  | 317229097 | FABP1 |
|  | 317229097 | PTGS2 |
|  | 317229097 | NCSTN |
|  | 317229097 | JAK1 |
| 40 | 249925971 | RORC |
|  | 249925971 | FABP1 |
|  | 249925971 | NCSTN |
|  | 249925971 | CNR2 |
|  | 249925971 | SCN9A |
|  | 249925971 | PTGS2 |
|  | 249925971 | JAK1 |
| 41 | 482043036 | RORC |
|  | 482043036 | FABP1 |
|  | 482043036 | NCSTN |
|  | 482043036 | JAK1 |
|  | 482043036 | PTGS2 |
|  | 482043036 | PTPN22 |
| 42 | 481106870 | CNR2 |
|  | 481106870 | ADORA1 |
|  | 481106870 | RORC |
| 43 | 482040418 | CNR2 |
|  | 482040418 | NCSTN |
|  | 482040418 | JAK1 |
|  | 482040418 | SCN9A |
|  | 482040418 | PIK3CD |
|  | 482040418 | PTPN22 |
| 44 | 482032650 | RORC |
|  | 482032650 | FABP1 |
|  | 482032650 | PTGS2 |
|  | 482032650 | NCSTN |
|  | 482032650 | JAK1 |
|  | 482032650 | CNR2 |
|  | 482032650 | PTPN22 |

**Table 4. Targets and Their Associated Pathways**

| **Target** | **Pathway** |
| --- | --- |
| CASP8 | Necroptosis |
| PARP1 | Necroptosis |
| IL1B | Necroptosis |
| EIF2AK2 | Necroptosis |
| PLA2G4A | Necroptosis |
| JAK1 | Necroptosis |
| ALK | Pathways in cancer |
| NQO1 | Pathways in cancer |
| JUN | Pathways in cancer |
| CASP8 | Pathways in cancer |
| PTGS2 | Pathways in cancer |
| MTOR | Pathways in cancer |
| JAK1 | Pathways in cancer |
| NFE2L2 | Pathways in cancer |
| JUN | Kaposi sarcoma-associated herpesvirus infection |
| CASP8 | Kaposi sarcoma-associated herpesvirus infection |
| EIF2AK2 | Kaposi sarcoma-associated herpesvirus infection |
| PTGS2 | Kaposi sarcoma-associated herpesvirus infection |
| MTOR | Kaposi sarcoma-associated herpesvirus infection |
| JAK1 | Kaposi sarcoma-associated herpesvirus infection |
| ALK | PD-L1 expression and PD-1 checkpoint pathway in cancer |
| JUN | PD-L1 expression and PD-1 checkpoint pathway in cancer |
| LCK | PD-L1 expression and PD-1 checkpoint pathway in cancer |
| MTOR | PD-L1 expression and PD-1 checkpoint pathway in cancer |
| JAK1 | PD-L1 expression and PD-1 checkpoint pathway in cancer |
| JUN | Th17 cell differentiation |
| LCK | Th17 cell differentiation |
| IL1B | Th17 cell differentiation |
| MTOR | Th17 cell differentiation |
| JAK1 | Th17 cell differentiation |
| JUN | TNF signaling pathway |
| CASP8 | TNF signaling pathway |
| IL1B | TNF signaling pathway |
| PTGS2 | TNF signaling pathway |
| SELE | TNF signaling pathway |
| NQO1 | Fluid shear stress and atherosclerosis |
| JUN | Fluid shear stress and atherosclerosis |
| IL1B | Fluid shear stress and atherosclerosis |
| SELE | Fluid shear stress and atherosclerosis |
| NFE2L2 | Fluid shear stress and atherosclerosis |
| JUN | Measles |
| CASP8 | Measles |
| IL1B | Measles |
| EIF2AK2 | Measles |
| JAK1 | Measles |
| JUN | Leishmaniasis |
| IL1B | Leishmaniasis |
| PTGS2 | Leishmaniasis |
| JAK1 | Leishmaniasis |
| JUN | Lipid and atherosclerosis |
| CASP8 | Lipid and atherosclerosis |
| IL1B | Lipid and atherosclerosis |
| SELE | Lipid and atherosclerosis |
| NFE2L2 | Lipid and atherosclerosis |
| CASP8 | Human cytomegalovirus infection |
| IL1B | Human cytomegalovirus infection |
| PTGS2 | Human cytomegalovirus infection |
| MTOR | Human cytomegalovirus infection |
| JAK1 | Human cytomegalovirus infection |
| JUN | IL-17 signaling pathway |
| CASP8 | IL-17 signaling pathway |
| IL1B | IL-17 signaling pathway |
| PTGS2 | IL-17 signaling pathway |
| JUN | C-type lectin receptor signaling pathway |
| CASP8 | C-type lectin receptor signaling pathway |
| IL1B | C-type lectin receptor signaling pathway |
| PTGS2 | C-type lectin receptor signaling pathway |
| PARP1 | NF-kappa B signaling pathway |
| LCK | NF-kappa B signaling pathway |
| IL1B | NF-kappa B signaling pathway |
| PTGS2 | NF-kappa B signaling pathway |
| JUN | Osteoclast differentiation |
| LCK | Osteoclast differentiation |
| IL1B | Osteoclast differentiation |
| JAK1 | Osteoclast differentiation |
| CASP8 | Human papillomavirus infection |
| EIF2AK2 | Human papillomavirus infection |
| PTGS2 | Human papillomavirus infection |
| MTOR | Human papillomavirus infection |
| JAK1 | Human papillomavirus infection |
| CASP8 | Alzheimer disease |
| IL1B | Alzheimer disease |
| EIF2AK2 | Alzheimer disease |
| PTGS2 | Alzheimer disease |
| MTOR | Alzheimer disease |
| CASP8 | Influenza A |
| IL1B | Influenza A |
| EIF2AK2 | Influenza A |
| JAK1 | Influenza A |
| JUN | NOD-like receptor signaling pathway |
| CASP8 | NOD-like receptor signaling pathway |
| IL1B | NOD-like receptor signaling pathway |
| JAK1 | NOD-like receptor signaling pathway |
| JUN | Epstein-Barr virus infection |
| CASP8 | Epstein-Barr virus infection |
| EIF2AK2 | Epstein-Barr virus infection |
| JAK1 | Epstein-Barr virus infection |
